# A nacre-inspired structural material with thermochromic properties and mechanical robustness by atomic-level design

**DOI:** 10.1093/nsr/nwaf098

**Published:** 2025-03-17

**Authors:** Jun Pang, Ze-Yu Wang, Tao Song, Zhen-Bang Zhang, Yu-Feng Meng, Si-Chao Zhang, Long Zhang, Wei-Yi Xing, Shu-Hong Yu

**Affiliations:** New Cornerstone Science Laboratory, Department of Chemistry, Institute of Biomimetic Materials & Chemistry, Anhui Engineering Laboratory of Biomimetic Materials, Division of Nanomaterials & Chemistry, Hefei National Research Center for Physical Sciences at the Microscale, University of Science and Technology of China, Hefei 230026, China; New Cornerstone Science Laboratory, Department of Chemistry, Institute of Biomimetic Materials & Chemistry, Anhui Engineering Laboratory of Biomimetic Materials, Division of Nanomaterials & Chemistry, Hefei National Research Center for Physical Sciences at the Microscale, University of Science and Technology of China, Hefei 230026, China; School of Information Science and Technology, University of Science and Technology of China, Hefei 230026, China; Department of Chemistry, Institute of Innovative Materials, Shenzhen Key Laboratory of Sustainable Biomimetic Materials, Guangming Advanced Research Institute, Southern University of Science and Technology, Shenzhen 518055, China; New Cornerstone Science Laboratory, Department of Chemistry, Institute of Biomimetic Materials & Chemistry, Anhui Engineering Laboratory of Biomimetic Materials, Division of Nanomaterials & Chemistry, Hefei National Research Center for Physical Sciences at the Microscale, University of Science and Technology of China, Hefei 230026, China; New Cornerstone Science Laboratory, Department of Chemistry, Institute of Biomimetic Materials & Chemistry, Anhui Engineering Laboratory of Biomimetic Materials, Division of Nanomaterials & Chemistry, Hefei National Research Center for Physical Sciences at the Microscale, University of Science and Technology of China, Hefei 230026, China; New Cornerstone Science Laboratory, Department of Chemistry, Institute of Biomimetic Materials & Chemistry, Anhui Engineering Laboratory of Biomimetic Materials, Division of Nanomaterials & Chemistry, Hefei National Research Center for Physical Sciences at the Microscale, University of Science and Technology of China, Hefei 230026, China; State Key Laboratory of Fire Science, University of Science and Technology of China, Hefei 230026, China; New Cornerstone Science Laboratory, Department of Chemistry, Institute of Biomimetic Materials & Chemistry, Anhui Engineering Laboratory of Biomimetic Materials, Division of Nanomaterials & Chemistry, Hefei National Research Center for Physical Sciences at the Microscale, University of Science and Technology of China, Hefei 230026, China; Department of Chemistry, Institute of Innovative Materials, Shenzhen Key Laboratory of Sustainable Biomimetic Materials, Guangming Advanced Research Institute, Southern University of Science and Technology, Shenzhen 518055, China

**Keywords:** biomimetic structural materials, mechanical robustness, thermochromic properties, active early fire warning, passive flame retardancy

## Abstract

Advanced structural materials are often required to exhibit a combination of light weight, high strength and superior toughness. Biomimetic strategies hold promise for achieving these seemingly conflicting mechanical properties simultaneously. However, current biomimetic structural materials lack active fire-warning and passive flame-retardant functionalities, which poses risks for their application in fire-prone scenarios. Herein, we present a nacre-mimetic alumina–cyanate resin composite (NAC) that has a combination of mechanical robustness with thermochromic and flame-retardant properties. Through controlled atomic doping, chromium atoms are incorporated into alumina microplatelets, forming solid-solution assembly units that exhibit reversible thermochromism and a solid-solution-strengthening effect. The bioinspired ‘brick-and-mortar’ structure endows the NAC with high strength (∼290.1 MPa) and fracture toughness (∼11.1 MPa m^1/2^). Coupled with a machine-learning-based image-recognition system, the NAC leverages its thermochromic properties to deliver a rapid fire warning within 9 s at 250°C, which is significantly faster than traditional electronic fire alarms. Its layered structure effectively impedes oxygen flow, achieving an oxygen-limiting index of 50%, and thus ensuring excellent flame-retardant performance. This design delays the combustion peak and reduces the heat-release value, thereby enhancing the flame-retardant performance. This work demonstrates the effective integration of a structural and functional design for active early fire warning and passive flame retardancy, paving the way for structural materials in advanced fire-warning systems in challenging environments.

## Teaser

By employing atomic-level doping strategies, the bioinspired nacre-mimetic structural material achieves the effective integration of thermochromic function and mechanical robustness for active early fire warning and passive flame retardancy.

## INTRODUCTION

Advanced structural materials are extensively utilized in fields such as aerospace, construction and automotive manufacturing, in which they are often required to exhibit a combination of light weight, high strength and superior toughness [1–4]. Mimicking of the complex hierarchical structures of natural biomaterials has been shown to achieve these seemingly conflicting mechanical properties [5,6]. For instance, replicating the highly ordered organic–inorganic ‘brick-and-mortar’ (BM) microstructure and the reinforcing effect of mineral bridges that is found in nacre can result in structural materials with enhanced strength and toughness [5,7–10]. As the demand for multifunctional applications continues to grow, these materials must also possess additional functionalities to adapt to complex environmental changes [11,12]. In certain fire-prone scenarios, for example, the high temperatures that are generated by uncontrolled fires can significantly degrade the mechanical performance of structural materials, posing risks of equipment failure or structural collapse [13]. However, the inherent limited thermal resistance of the organic components in biomimetic composite materials constrains their overall high-temperature performance. This necessitates the addition of fire-alarm and flame-retardant systems, which may compromise reliability due to increased complexity [13–15]. Therefore, it is of great practical significance to develop structural materials with both active early fire-warning and passive flame-retardant properties.

It is crucial to select active fire-warning and passive flame-retardant mechanisms that can be integrated with the intrinsic properties of structural materials. Recently, thermochromic materials combined with image-recognition systems have demonstrated promising potential in early fire-warning applications [16]. By leveraging the thermochromic response that is triggered by high-temperature heat sources, these systems can generate immediate alarm signals, facilitating timely detection and response to fire hazards [16–18]. Compared with traditional fire-warning systems, such as smoke alarms, this approach addresses issues of low sensitivity, delayed response times and high false-alarm rates [14,19–21]. Additionally, these systems do not rely on external electrical circuits, thereby reducing the impact of complex environments on their performance [14,16]. For passive flame-retardant mechanisms, the aforementioned nacre-inspired BM microstructure has been demonstrated to possess inherent fire-prevention and flame-retardant capabilities [22–24]. The primary flame-retardant mechanism is attributed to the barrier effect of the lamellae, which impedes heat transfer, the diffusion of pyrolysis products and the mixing of oxygen [25,26]. However, at this stage, the incorporation of thermochromic functionality into structural materials presents a significant challenge.

Herein, we propose a strategy that combines an atomic-doping design with a biomimetic structural design to prepare nacre-mimetic alumina–cyanate resin composites (NACs) with mechanical robustness, thermochromic properties and flame-retardant functionality. Through a controlled solid-solution reaction, chromium-doped alumina microplatelets (Cr-doped Al_2_O_3_ MPs) are synthesized to serve as ‘bricks’ in the nacre-inspired BM structure. These microassembly units exhibit unique reversible thermochromic properties. Simultaneously, this atomic-doping strategy facilitates solid-solution strengthening [27–30], which synergizes with the BM structural design, endowing the NAC with light weight (∼2.3 g cm^–3^), high flexural strength (∼290.1 MPa) and high fracture toughness (∼11.1 MPa m^1/2^). Moreover, the NAC maintains 62.8% flexural mechanical strength (∼182.4 MPa) at 250°C, significantly surpassing that of layered ceramic scaffolds (∼34.8 MPa) due to the infiltration of high-temperature-resistant cyanate resin (CE). Through image-recognition that is based on the trained K-means model [31,32], the response time of the NAC is 9 s at 250°C for the early fire warning. In addition, the highly ordered BM structure effectively obstructs oxygen conduction, achieving an oxygen-limiting index of 50%, and thus imparting excellent flame-retardant properties to the NAC. This mechanically robust bioinspired composite integrates active early fire-warning and passive flame-retardant strategies, making it a promising smart structural material for fire-warning systems in various harsh environments.

## RESULTS AND DISCUSSION

### Design and fabrication of NACs

The integration of microstructured building blocks with thermochromic properties into a nacre-inspired BM structure is a fundamental approach for constructing NACs. We propose a strategy that combines an atomic-doping design with a biomimetic structural design to realize this vision. Cr^3+^ exhibits different electronic transition phenomena in its d orbitals at varying temperatures [33–35]. The thermochromic properties of 2D microassembly units can be achieved through controlled Cr^3+^ doping. Additionally, the solid-solution strengthening effect enhances the mechanical strength of these Al_2_O_3_ MPs. By utilizing these thermochromic Cr-doped Al_2_O_3_ MPs as ‘bricks’ and the high-temperature-resistant polymer as the ‘mortar’, we aim to construct a nacre-inspired BM structure that simultaneously achieves excellent mechanical performance and flame retardancy in NACs.

**Figure 1.**
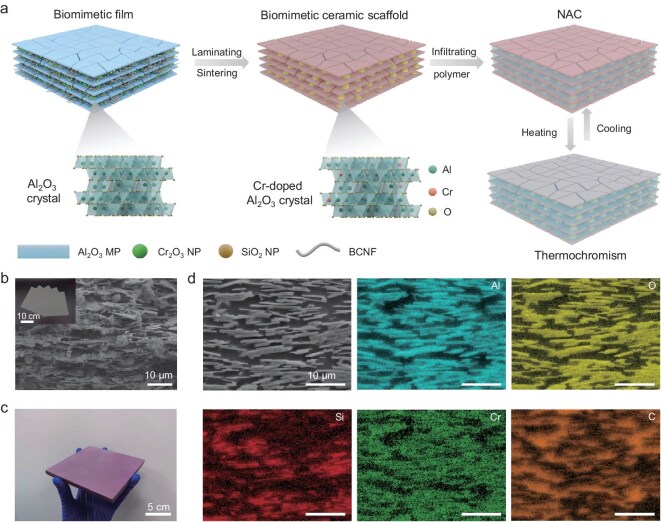
Fabrication and microstructure characterization of the NAC. (a) Schematic illustration of multiscale structure design and fabrication of the biomimetic bulk composite. (b) Cross-sectional scanning electron microscope (SEM) image of the layered nanocomposite film. The upper-left insert shows a photograph of the nanocomposite films. (c) Photograph of the NAC. (d) SEM elemental maps of the NAC.

Specifically, we prepared NACs through a bottom-up assembly process, as illustrated in Fig. 1a. Nacre-inspired films were produced by using evaporation-induced self-assembly. This process employed Al_2_O_3_ MPs, silicon dioxide nanoparticles (SiO_2_ NPs) and chromium oxide nanoparticles (Cr_2_O_3_ NPs) as inorganic components, with bacterial cellulose nanofibers (BCNFs) serving as the organic component (Fig. 1b and Fig. S1). The BCNFs network trapped Al_2_O_3_ MPs and NPs, forming homogeneous films (Fig. S2). The green nanocomposite films were laminated and sintered to construct layered ceramic scaffolds (Fig. S3). The sintered SiO_2_ NPs acted as mineral bridges connecting Al_2_O_3_ MPs, while Cr_2_O_3_ NPs reacting with Al_2_O_3_ MPs to form Cr-doped Al_2_O_3_ solid solution at a high temperature of 1500°C. Then, the ceramic scaffolds underwent surface treatment with *N-*[3-(trimethoxysily)propyl]ethylenediamine (Z6020) (Fig. S4). After infiltration with CE, we obtained a densified, multiscale-structured bulk composite (Fig. 1c and Fig. S5a). The orientation degree of Cr-doped Al_2_O_3_ MPs in the bioinspired bulk composite was quantified as 89.9% by using 2D small-angle X-ray scattering analysis (Fig. S5b), indicating a high degree of orientation in the NACs. As shown in Fig. 1d, the distribution of individual elements in the bulk composite reveals that the elemental Si is located between the elemental Al, confirming the successful formation of mineral bridges between Al_2_O_3_ MPs. Additionally, the overlap of the elemental Cr with the elemental Al preliminarily proves the formation of the solid solution (Fig. 1d).

### Thermochromic mechanism of NACs

**Figure 2.**
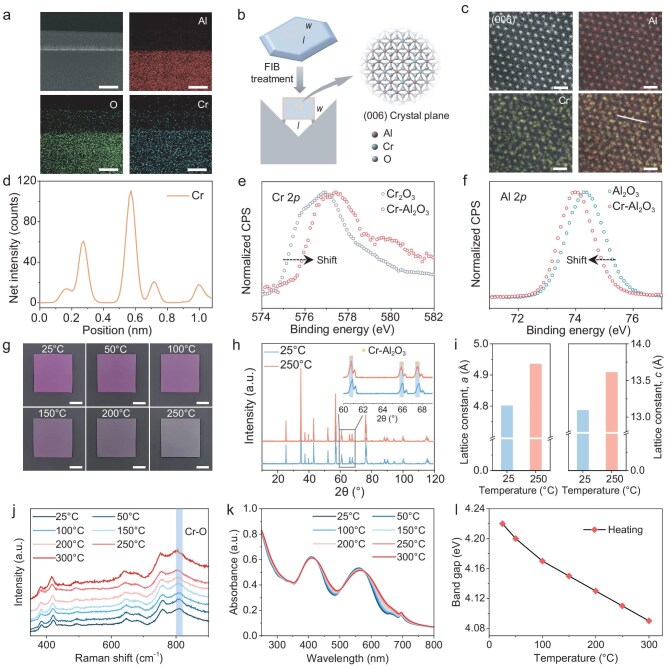
Thermochromic mechanism of NACs. (a) TEM elemental maps of the cross-sectional Cr-doped A_2_O_3_ MP. Scale bars, 200 nm. (b) Illustration of the Cr-doped Al_2_O_3_ MP cut by FIB parallel to the *c*-axis of the crystal shows the (006) crystal plane. (c) Atomic-resolution HAADF-STEM image and the corresponding EDS mapping of the Cr-doped Al_2_O_3_ MP, taken with the *c*-axis of the crystal parallel to the electron beam. Scale bars, 0.2 nm. (d) Intensity profiles of elemental Cr along the white line in (c). (e and f) High-resolution XPS spectra of (e) Cr 2*p* and (f) Al 2*p*. (g) Photographs of the thermochromic NAC from 25°C to 250°C. Scale bars, 1 cm. (h) *In situ* heated HRXRD profiles of Cr-doped Al_2_O_3_ MPs powder. (i) Lattice parameters of the Cr–Al_2_O_3_ solid solution at 25°C and 250°C. (j) *In situ* heated Raman spectra and (k) UV–vis absorption spectra of the Cr-doped layered ceramic scaffold. (l) Calculated band gap of the Cr-doped layered ceramic scaffold from Tauc's relationship as a function of the temperatures of the Cr-doped layered ceramic scaffold.

The thermochromic properties of NACs are attributed to the atomic-doping design of the microassembly units. To characterize the atomic structure of Cr-doped Al_2_O_3_ MPs, we mixed Al_2_O_3_ MPs and Cr_2_O_3_ NPs at a weight ratio of 1:0.1, consistently with that used in NACs, and then annealed them at 1500°C. Notably, the MPs powder turned pinkish after annealing (Fig. S6). Furthermore, we cut the Cr-doped Al_2_O_3_ MP by using a focused ion beam (FIB) and observed Cr diffusion into the MP (Fig. 2a and Fig. S7). High-resolution transmission electron microscopy (HRTEM) images revealed that the lattice of the Cr-doped Al_2_O_3_ MP was slightly larger than that of the undoped Al_2_O_3_ MP (Fig. S8). To better observe the Cr monoatomic state, we determined that the crystal plane that was parallel to the surface of the single-crystal Al_2_O_3_ MP was the (006) crystal plane (Fig. S9). The transmission electron microscopy (TEM) sample was then prepared from vertical (006) crystal planes (Fig. 2b), parallel to the *c*-axis of the crystal, by using the FIB technique [36]. We employed aberration-corrected (AC) high-angle annular dark-field scanning transmission electron microscopy (HAADF-STEM) with energy-dispersive X-ray spectroscopy (EDS) to reveal the precise location of the Cr atoms in the Cr-Al_2_O_3_ solid solution, in which two high-density bright spots corresponded to Cr single atoms (Fig. 2c and Fig. S10). The integrated pixel intensity profiles further confirmed the substitution of Al single atoms with Cr single-atom dopants (Fig. 2d and Fig. S11). Furthermore, the shift of the Cr 2*p* peak in the X-ray photoelectron spectroscopy (XPS) spectra verified the different chemical environments for Cr atoms before and after doping (Fig. 2e). To determine specific substitution sites, we analysed the high-resolution XPS spectra of Al 2*p* and O 1*s*. The Al 2*p* peaks of the doped sample shifted towards lower binding energies (binding energy difference ∼0.3 eV) (Fig. 2f), suggesting that the Cr substitution for Al strengthened the average ionicity between the Al and the O in the doped sample [37]. Additionally, the binding energy of O also decreased by ∼0.3 eV compared with the undoped sample, attributed to the donor-doping effect [38,39] induced by the Cr, which donates extra electrons to the system (Fig. S12). Therefore, the incorporation of Cr atoms significantly altered the chemical environments within the Al_2_O_3_ crystal.

By using Cr-doped Al_2_O_3_ MPs as the assembly building blocks, we successfully endowed layered ceramic scaffolds and biomimetic bulk composites with thermochromic properties (Fig. 2g and Fig. S13). X-ray diffraction (XRD) spectra revealed peaks of a new phase near the Al_2_O_3_ crystal peaks, attributed to a Cr-doped Al_2_O_3_ solid-solution crystal [40] (Fig. S14a–c). Meanwhile, the addition of SiO_2_ to the ceramic scaffolds had no effect on this crystal phase change (Fig. S14d). To explore the thermochromic mechanism, we utilized *in situ* heating high-resolution XRD (HRXRD) to obtain the diffraction peaks shift data of the Cr-doped Al_2_O_3_ MPs powder. The diffraction peaks shifted to lower angles (Fig. 2h), indicating lattice expansion with increasing temperature. The change in the lattice parameters at 25°C and 250°C were calculated by using the Rietveld refinement method [41,42] to validate this observation (Fig. 2i and Fig. S15). The colors of the transition-metal complexes are derived from electronic transitions between d orbitals that are split by the ligand field [35]. This splitting of the d orbitals is also the origin of the red color in the Cr-doped Al_2_O_3_ [35]. At high temperatures, lattice expansion reduces the constraint of the O^2^^–^ anions on the Cr^3+^ valence orbitals, allowing the material to regain its original green color based on the ligand field theory of transition-metal complexes [35,43]. Furthermore, the temperature-dependent evolution of the bond length and the lattice dimension was investigated by using *in situ* Raman spectroscopy from 25°C to 300°C upon heating. The Cr–O stretching vibration exhibited an intensity peak at 810 cm^–1^ (Fig. S16a and b). As the temperature increased, the Cr–O stretching vibration intensity peak redshifted (Fig 2j and Fig. S16c). This was attributed to Cr–O bond elongation, further demonstrating the temperature-dependent evolution of the bond length and lattice dimensions for color change [34,44]. In addition, ultraviolet-visible (UV–vis) absorption spectroscopy was carried out at different temperatures to quantitatively characterize the thermochromic properties. The absorption spectra (Fig. 2k and Fig. S17a) revealed two broad bands in the visible range, at 370–430 and 530–580 nm. These bands are associated with the d–d electronic transitions of Cr^3+^: ^4^A_2__g_ → ^4^T_1__g_ (370–430 nm) and ^4^A_2__g_ → ^4^T_2__g_ (530–580 nm) (Fig. S17b–d) [45]. The ligand field theory for Cr^3+^ in an octahedral environment predicts the existence of three absorption bands [45]. The energies of the first two electronic spins allowed the transitions ^4^A_2__g_ → ^4^T_1g_(F) and ^4^A_2__g_ → ^4^T_2g_(F), which corresponded to visible light [43,45], whereas the third spin allowed the transition from ^4^A_2__g_ to ^4^T_1g_(P), corresponding to ultraviolet light, which does not affect the color [43,45]. The ligand field that was created by the oxide ions allows adjustment of the positions of these absorption bands, resulting in different colors at varying temperatures (Fig. 2k). Moreover, the optical band gap of the layered ceramic scaffolds was determined by extrapolating the linear region of the absorption edge to the energy–axis intercept (Fig. S18). As the temperature increased from 25°C to 300°C, the optical band gap reduced from 4.22 to 4.09 eV (Fig. 2l). This narrowing of the band gap was likely due to deformation of the Cr–O polyhedron and consistent with the observed color change from pinkish to gray as the temperature rose [34,44,46].

### Mechanical properties of NACs

**Figure 3.**
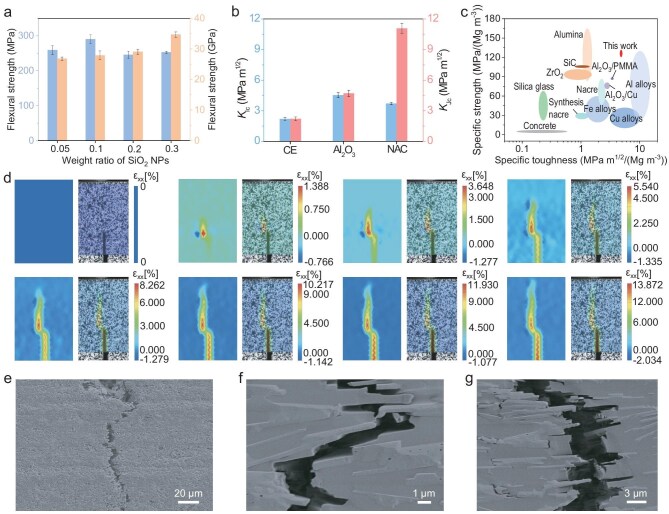
Macromechanics of biomimetic bulk structural materials. (a) Flexural strength and Young's modulus of NACs. (b) Fracture toughness for crack initiation (*K*_Ic_) and stable crack propagation (*K*_Jc_) of NACs, CE and Al_2_O_3_ ceramics. (c) Ashby diagram of specific strength and specific toughness for NACs compared with a series of engineering materials. (d) DIC maps of the NAC during the crack propagation. (e) Long-range crack deflection. (f) Relative displacement of Al_2_O_3_ MPs derived from shear loading. (g) Al_2_O_3_ MPs pull-out with crack growth.

The hierarchical architectures of nacre-mimetic materials play a dominating role in their exceptional mechanical properties. To evaluate the effectiveness of the multiscale structure, we systematically studied the mechanical properties of NACs. We selected a weight ratio of Cr_2_O_3_ NPs to Al_2_O_3_ MPs of 1:0.1, considering the significant color change and high mechanical strength at this ratio (Figs S19 and S20). Subsequently, by altering the content of the SiO_2_ NPs, we achieved an optimal flexural strength of ∼290.1 MPa for the NAC (Fig. 3a and Fig. S21). This is mainly due to the optimization of the layered orientation degree of the bioinspired composite at the microscopic level (Fig. S22). Moreover, the number of mineral bridges connecting the Cr-doped Al_2_O_3_ MPs increased with the weight ratio of the SiO_2_ NPs, which also led to enhanced bending strength of the layered ceramic scaffolds (Fig. S23a). Besides, the inorganic content of the NACs increased (Fig. S23b). These factors also contributed to the increased modulus of the bioinspired bulk composites (Fig. 3a). Furthermore, comparison of the flexural strength of the layered ceramic scaffolds without SiO_2_ and Cr_2_O_3_ NPs incorporation confirmed that sintered SiO_2_, which connects Cr-doped Al_2_O_3_ MPs, significantly contributes to the mechanical support of layered ceramic scaffolds (Fig. S24). In addition, the optimal NAC exhibited advantages in mechanical strength and specific strength compared with engineering Al_2_O_3_ ceramics and synthetic CE (Fig. S25).

The fracture toughness *K*_Ic_, indicating resistance to crack initiation, was ∼3.7 MPa m^1/2^ for the optimal NAC, which was slightly lower than that of engineering Al_2_O_3_ ceramics (∼4.5 MPa m^1/2^) but higher than that of CE (∼2.19 MPa m^1/2^) (Fig. 3b). The maximum fracture toughness *K*_Jc_ of the NAC (∼11.1 MPa m^1/2^) was measured to be approximately three times higher than the initial resistance value, far exceeding those of engineering Al_2_O_3_ ceramics (∼4.7 MPa m^1/2^) and CE (∼2.21 MPa m^1/2^) (Fig. 3b). These results illustrate that the high strength and toughness of the bioinspired composite are attributed to its hierarchical structure. Figure 3c and Table S1 show that the specific strength and specific toughness of the hierarchical NAC surpass those of various engineering materials [47–49].

Multiple extrinsic toughening mechanisms effectively resist crack growth, primarily operating in the crack wake [5]. These mechanisms were further investigated via fracture mechanics analysis. The fracture resistance and deformation mechanisms were evaluated by using the notched NAC and confirmed by 2D digital image correlation (DIC). A significant deflection phenomenon was observed from crack initiation to expansion, with the strain level at failure reaching as high as 13.87% (Fig. 3d and e). Besides, X-ray tomography imaging showed that specimen cracks propagated across different planes (Fig. S26). Those results proved that the fracture propagation process of the NAC was stable and long-lasting, and exhibited characteristics of quasi-plastic fracture [50,51]. In contrast, CE and engineering Al_2_O_3_ ceramics did not exhibit significant crack deflection due to their isotropic and homogeneous structure (Figs S27 and S28). Furthermore, the failure strain of the Al_2_O_3_ ceramic was only 1.59%, indicating that it was a brittle fracture (Fig. S28). In addition, the state of crack growth was observed by using a combination of a high digital camera and an optical microscope. The structural design of the NAC can effectively prevent crack growth and prolong the path and duration of crack growth (Movies S1–S3). Higher-magnification SEM images illustrate the relative displacement of individual MPs and their pull-out from the organic phase due to local tensile stress and interfacial shear stresses (Fig. 3f and g, and Fig. S29), which are important micromechanisms for energy absorption to improve toughness [52,53]. These results confirm that the multiscale microstructure contributes to the load redistribution and toughness enhancement of NACs.

**Figure 4.**
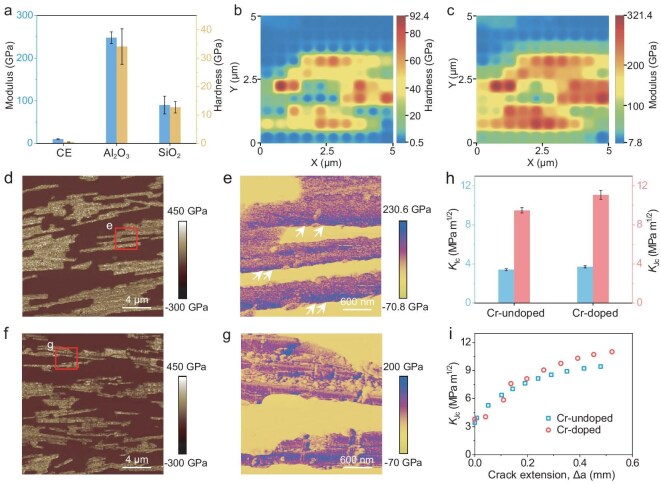
Micromechanics of NACs related to microstructure. (a) Microstructure modulus and hardness of NACs. (b and c) Maps of the (b) hardness and (c) modulus of the NAC. (d) Elastic modulus map of the NAC based on AFM measurements. (e) Magnifying elastic modulus map in (d). (f) Elastic modulus map of the NAC without doped Cr_2_O_3_ NPs. (g) Magnifying elastic modulus map in (f). (h) Comparison of facture toughness of NACs with doped and undoped Cr_2_O_3_ NPs. (i) *R*-curves showing the resistance to fracture in terms of the stress intensity, *K*_Jc_, as a function of the crack extension, *Δ*a, for NACs with doped and undoped Cr_2_O_3_ NPs.

To further explore the microscopic mechanics of the NAC, we conducted nanoindentation tests to measure the modulus and hardness of its components. The modulus of Cr-doped Al_2_O_3_ MPs, CE and SiO_2_ mineral bridges were measured as ∼248.0, ∼10.2 and ∼90.0 GPa, while their hardness was tested as ∼34.07, ∼0.50 and ∼12.72 GPa, respectively (Fig. 4a and Fig. S30). The stacked microstructure, composed of alternating soft and hard phases, similarly to natural nacre, is a key factor in achieving the light weight, high strength and high toughness of the NAC (Fig. 4b and c) [5,54,55]. To better detect the interface mechanics of Cr-doped Al_2_O_3_ MPs, we conducted atomic force microscopy (AFM) to obtain modulus mapping (Fig. 4d). From the magnification modulus mapping, we found that the modulus of the Cr-doped Al_2_O_3_ MPs interface was higher than that of the Al_2_O_3_ MPs themselves, due to the solid-solution strengthening effect (Fig. 4e and Fig. S31). Moreover, this effect facilitates the reduction of transgranular fractures in brittle inorganic MPs, thereby enhancing toughness [56,57]. In contrast, in the composites without doped Cr_2_O_3_ NPs, the modulus of the Al_2_O_3_ MPs was the highest, causing them to rupture more easily compared with Cr-doped MPs during crack propagation (Fig. 4f and g). Furthermore, the fracture toughness of the NACs was higher than that of undoped ones (Fig. 4h). Simultaneously, the extensive rising crack-resistance curves (*R*-curves) exhibited behavior that was similar to those of natural nacre [5], indicating enhanced resistance to fracture during crack propagation (Fig. 4i). These results demonstrate that the atomic-doping design not only imparts thermochromic properties to NACs, but also enhances their fracture toughness.

### NACs for the early fire-warning system

**Figure 5.**
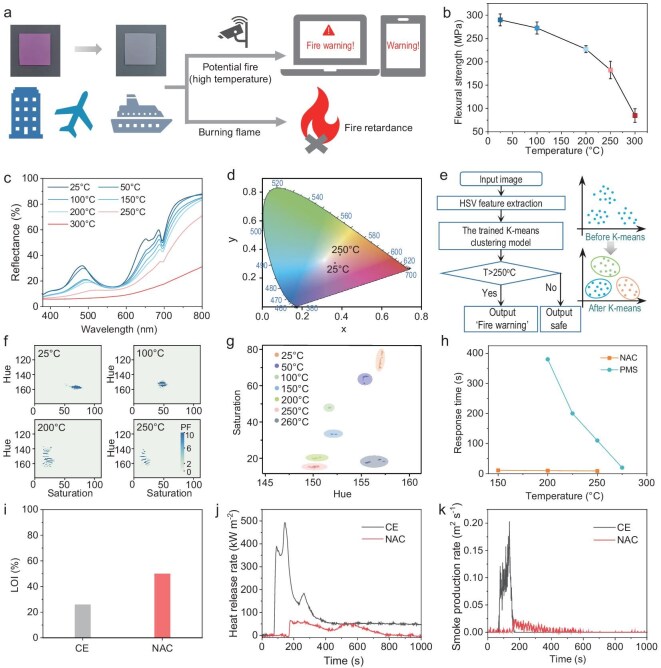
Application of NACs for the early fire-warning system. (a) Schematic illustration of NACs for the active early fire warning and passive fire retardance. (b) Ambient flexural strength of NACs at different temperatures. (c) Reflectance spectra of the NAC under different temperatures. (d) International commission on illumination (CIE) color coordinates map of NACs at 25°C and 250°C. (e) Color information processing based on K-means model. (f) 2D H–S color histograms of the NAC at different temperatures. The color bar values represent pixel frequency. (g) Clustering of images based on H–S features. (h) Comparison of response time of the NAC with the thermochromic material reported in reference (PMS refers to phthalocyanines precursor molecular sensor) [16]. (i–k) Comparison of flame retardance of the NAC and CE in terms of LOI (i), heat-release rate (j) and smoke-production rate (k).

Ideal thermochromic materials for early fire warning should exhibit sensitive thermochromic responses prior to flame emergence and effective flame-retardant properties thereafter (Fig. 5a). The ignition temperatures of common combustibles in a fire range from 250°C to 500°C [13]. Therefore, it is crucial for NACs to ensure a thermochromic response and mechanical stability below the ignition temperatures of various combustible polymeric materials. As shown in Fig. S32a, the dimensions of the NAC and ceramic scaffold showed minimal variation with temperature whereas CE exhibited significant changes as the temperature increased. Due to the anisotropy of the layered structure, the out-of-plane thermal conductivity improved significantly following resin infiltration (Fig. S32b). This enhancement facilitates heat dissipation, thereby accelerating the speed of color change. Furthermore, we conducted dynamic mechanical analysis (DMA) to explore the storage modulus, loss modulus and mechanical loss angle (tanδ) as the temperature increased at a frequency of 1 Hz. After CE was infiltrated into the layered ceramics to form NACs, both the storage energy modulus and loss modulus decreased as the temperature increased, with the peak tanδ value occurring at 276°C (Fig. S33). This indicates that temperatures of ≤250°C, which are exactly under the ignition temperatures of common combustibles for fire warnings, can be considered the safe operating range for NACs. The ambient bending strength of NACs decreased as the temperature increased (Fig. 5b). They can be employed as engineering materials with a flexural strength of 182.4 MPa at 250°C (Fig. 5b). However, the bending strength decreased significantly to 84.9 MPa at 300°C, posing threats to practical use (Fig. 5b). Meanwhile, the color change of the NAC corresponded to the trend observed in crystal heating color changes at temperatures of <250°C but deviated from this trend at 300°C (Fig. 5c and Figs S34 and S35). There is a distinct color difference in the NAC at 25°C and 250°C (Fig. 5d), making it suitable for early fire-warning signals.

To describe the color change quantitatively, the HSV (Hue, Saturation, Value) color model is employed to transform the information into a recognizable format by the computer. By extracting the HSV features of image colors at different temperatures, the trained K-means clustering model is used to process and classify color information (Fig. 5e). Figure 5f and Figs S36 and S37 show the 2D color histograms that were extracted at different temperatures, corresponding to the H and S values. Furthermore, the H–S features of the NACs were analysed by clustering at different temperatures (Fig. 5g). Temperatures of >250°C were defined as hazardous and the input of an image with colors that corresponded to these temperatures triggered a fire warning (Supplementary Movie S4). Moreover, we put NACs on the heat surface to simulate the high temperature that may occur during the precombustion phase of a fire-response process. The early-warning temperature thresholds are associated with specific colors, corresponding to defined temperature ranges. The sensitivity was then evaluated by measuring the time required for NACs to change color. By utilizing the trained image-recognition algorithm, the response time at various temperatures is determined based on the categorized HSV color features. Thus, the response time of the NAC is 11, 10 and 9 s at 150°C, 200°C and 250°C, respectively, demonstrating its high sensitivity at warning temperatures (Fig. 5h) compared with another thermochromic material that was reported in the literature [16]. Then, the accuracy of the image-recognition program was verified by using a thermal infrared camera (Figs S38–S40). Therefore, thermochromic NACs combined with intelligent image-recognition technology provide rapid and accurate monitoring for early fire-warning detection.

Besides active fire warning, NACs possess passive fire-prevention and flame-retardant functions under high-temperature ablation during the later stage of fire. The limiting oxygen index (LOI) test shows that the LOI value of NACs is 50%, compared with 26% for CE, attributed to the barrier effect of their layered structure (Fig. 5i). Furthermore, we utilize cone calorimetry testing to study the combustion behavior of the NAC in a real fire scenario. After testing, the NAC maintained its morphology integrity whereas the CE was fully carbonized (Fig. S41a). The peak heat-release rate of CE is ≤493 kW m^–2^ whereas that of the NAC decreases to 65 kW m^–2^ (Fig. 5j). Analysis of the heat-release rate and total heat-release curves indicates that delayed combustion peaks and decreased heat-release values enhance flame retardation (Fig. 5j and Fig. S41b). Simultaneously, the NAC releases significantly less smoke than CE, to enhance fire safety (Fig. 5k and Fig. S41c). Thus, highly ordered inorganic MPs in NACs not only provide excellent mechanical robustness, but also achieve superior flame retardance.

## CONCLUSION

In summary, the prepared NAC combines strong mechanical robustness with active early fire-warning and passive flame-retardant functions. During the sintering process, Cr^3+^ migrates into Al_2_O_3_ MPs, forming solid-solution assembly units. These units impart thermochromic properties to the NAC, enabling it to undergo rapid color changes in response to elevated temperatures. The bioinspired multiscale BM structure, enhanced by inorganic mineral bridges, synergistically improves the strength and toughness of the NAC. The solid-solution strengthening effect increases the interfacial modulus of Al_2_O_3_ MPs, reducing transcrystalline rupture and enhancing fracture toughness. By leveraging the thermochromic property, we utilize the trained K-means clustering model to process color variations digitally, enabling accurate and sensitive fire detection. Additionally, the layered structure provides effective flame retardance and reduces the generation of fire-related gases, addressing the later stages of combustion. This bioinspired design strategy provides a platform for creating structure–function integrated materials that are suitable for application in complex and dynamic environments.

## METHODS

### Materials

Al_2_O_3_ MPs with an average thickness of ∼400 nm and diameter size of ∼10 μm were purchased from Merck Co. Ltd, USA. SiO_2_ NPs and Cr_2_O_3_ NPs were purchased from Macklin. BCNFs dispersion was purchased from Guilin Qihong Technology Co., Ltd, China. Ethanol was purchased from Shanghai Sinopharm Group Co. Ltd, China. Z6020 was purchased from RHAWN. 2,2-Bis-(4-cyanatophenyl)propane was purchased from Wuhan Lullaby Pharmaceutical Chemical Co. Ltd, China.

### Characterization of the samples

XRD patterns of the ingredients and ceramic scaffolds powder were obtained by using an X-ray diffractometer (X’ PertMPD) with CuKα radiation (λ = 0.15406 nm). The XRD patterns of the ceramic scaffolds and nacre-mimetic composites were conducted by using high-resolution XRD for thin films (X’ Pert3 MRD). HRXRD patterns of Cr-doped Al_2_O_3_ MPs heated *in situ* were carried out on multifunctional rotating-anode XRD (SmartLab) at 0.02°/step and 10 s/step for data acquisition. The microstructure of the ceramic scaffolds and elemental maps of the NACs were observed by using SEM (Zeiss Genimi 450). The XPS (KRATOS, AXIS SUPRA+) was performed for elemental determination. HRTEM and TEM elemental maps of Al_2_O_3_ MPs were measured by using TEM (JEM ARM-200F) shooting after FIB (FEI, Helios NanoLab650) treatment. Atomic-resolution HAADF-STEM images and corresponding elemental maps were observed by using HRTEM (Themis Z) with a spherical aberration correction system. *In situ* heating Raman spectra were measured by using a laser confocal Raman microscope (LabRAM HR Evolution). UV–vis spectra were obtained by using a UV–vis-NIF spectrometer (SolidSpec-3700i DUV) with variable temperature attachments. The thickness of the Al_2_O_3_ MPs and the modulus of the bioinspired composites were characterized by using AFM (Bruker Dimension Icon). The modulus and hardness of the biomimetic composites were tested by using nanoindentation (Bruker Hysitron TI 980 TriboIndenter). The NAC was polished by using a grinding machine (Leica EM TXP) for the AFM and nanoindentation tests. Thermogravimetry analysis (TGA) of the biomimetic composites was conducted on a TA Q5000IR TGA from 25°C to 800°C with heating rate of 10°C min^–1^ under an air atmosphere. Fourier transform infrared spectroscopy (FTIR) spectra for surface treatment of the Z6020 were measured by using an FTIR spectrometer (Thermo Scientific Nicolet 8700). The 3D crack of the NAC after the toughness test was presented by using an X-ray microscope (Zeiss, Xradia 520 Versa). The dynamic crack propagation process of the biomimetic composites and comparative materials was captured by using a high-speed camera (Phantom VEO 1310 L) with an optical microscope (YueShi-YM710TR) on the *in situ* loading device (SD-Ra-98). The thermal conductivity of the layered ceramic scaffolds, CE and NACs was tested by using a laser thermal instrument (NETZSC LFA 467 LT). The thermal expansion of the NAC and its organic and inorganic components was performed via dilatometry (NETZSCH DIL 402 Expedis Supreme). DMA data of the NACs and CE were conducted by using a dynamic mechanical analyser (Discovery DMA850) from 25°C to 350°C. The LOI of the samples was measured by using oxygen index apparatus (HC-2) with samples of dimensions 100 × 10 × 4 mm^3^ according to the ASTM D2863 testing standard. Cone calorimetry experiments were conducted via a cone calorimeter (TESTECH EL3020) using ISO 5660 with a sample size of 100 × 100 × 3 mm^3^ under an external heat flux of 35 kW m^–2^.

## Supplementary Material

nwaf098_Supplemental_Files

## Data Availability

All Python scripts of code that were used in this study are available at https://github.com/lucasUSTC/CV_FireWarning.
