# Supplementary material for: A nacre-inspired structural material with thermochromic properties and mechanical robustness by atomic-level design
Source: Natl Sci Rev. 2025 Mar 17;12(5):nwaf098. doi: 10.1093/nsr/nwaf098 (PMC11983683; doi:10.1093/nsr/nwaf098)
Supplement: nwaf098_Supplemental_Files [file nwaf098_supplemental_files.zip › Supplementary data.pdf]

Supplementary Materials for

A nacre-inspired structural material with thermochromic properties and mechanical robustness by atomic-level design

Jun Pang[1,]†, Ze-Yu Wang[1,]†, Tao Song[2,]†, Zhen-Bang Zhang[3], Yu-Feng Meng[1], Si-Chao Zhang[1], Long Zhang[1], Wei-Yi Xing[4], Shu-Hong Yu[1,3,]*

Corresponding author: Shu-Hong Yu.
Email: shyu@ustc.edu.cn.

This file includes:
Supplementary text
Figures S1 to S41
Table S1

Other supplementary materials for this manuscript include the following:
Movies S1 to S4

Fabrication of the nanocomposite films

The weight ratio of Al_2O_3 MPs, SiO_2 NPs and Cr_2O_3 NPs was 1:0.1:0.1. The total 7.2g MPs and NPs were dispersed in 500 mL BCNFs dispersion solution of 0.08 wt.%. The mixture was dispersed by ultrasound for ~30 min and mechanically stirred for ~24 h to form a uniform suspension. Then, the vacuum pump was used to remove air bubbles from the suspension. After that, pour the green and homogeneous suspension into plastic culture dishes on heating platforms at 40℃. Finally, the layered nanocomposite films were obtained by water evaporation-induced self-assembly after ~24 h.

Fabrication of the bulk biomimetic composites

The cut layered nanocomposite films were laminated together through the hot press (10 MPa, 12 h) at room temperature to form layered bulk laminates. Then, the laminated bulks were put into a box furnace (Hefei Kejing Material Technology Co. Ltd., China) and sintered at 1500℃ for 6 h with the heating rate of 3℃ min^{-1}. After that, the obtained ceramic scaffolds were treated with coupling agent solution (Volume fraction ratio of Z6020: Ethanol 1:10) for 12 h at 60℃ to prepare for surface grafting treatment of platelets. Besides, 2,2-Bis-(4-cyanatophenyl)- propane was heated to 90℃ to melt into liquid. Then, the ceramic scaffolds were infiltrated easily by the vacuum in liquid 2,2-Bis-(4-cyanatophenyl)propane. So the air in the scaffolds pores replaced by liquid 2,2-Bis-(4-cyanatophenyl)propane. At last, the infiltrated ceramic scaffolds were placed in the oven and cured at 120℃ 1 h, 140℃ 1 h, 160℃ 1 h, 180℃ 3 h, 200℃ 1 h, 230℃ 3 h to obtain the bulk biomimetic composites.

SAXS characterization

The degree of orientation of Al_2O_3 MPs in bioinspired bulk composites was obtained by 2D SAXS (Anton Par SAXSpoint_2.0) experiments. The X-ray beam (Cu Kα radiation) was parallel to Al_2O_3 MPs of NACs with wavelength of 0.154 nm. The distance between the testing NAC and the detector was 567 mm. Fit2D software (the European Synchrotron Radiation Facility) was used to analyze the 2D SAXS data to acquire azimuthal angle (φ) plots. The degree of orientation of NACs is calculated as follows:

$$R = \frac{(180 - FWHM)}{180} \text{X}100$$

Where $FWHM$ is the full width at half maximum of the peak in the azimuthal intensity profile from azimuthal angle (φ) plots.

FIB treatment

Using the FIB system, the Al_2O_3 MPs were meticulously sectioned along the (006) crystal plane through plain-view imaging and lift-out methods. Subsequently, precise micro-manipulation was employed to securely mount the Al_2O_3 MPs onto a copper grid sample holder, ensuring that the (006) crystal plane was accurately aligned within the observation plane of the TEM. Thinning of the sample was then performed in a stepwise manner. When the thickness reached 50 nm, the voltage and beam current were reduced to 5 kV and 12 pA, respectively, to remove the amorphous surface layer. At 30 nm thickness, 2 kV and 5 pA were used for further surface refinement. Finally, at 20 nm thickness, 500 V and 48 pA were applied to completely eliminate the amorphous surface layer. The final ultrathin sample suitable for atomic-scale

observation was prepared using an argon ion thinning procedure.

Calculation of lattice parameters

The lattice of Cr-Al_2O_3 solid solution and Al_2O_3 crystal in Cr-doped Al_2O_3 MPs are obtained by HRXRD. The Rietveld refinement of HRXRD is performed by the GSAS software with the variation of pseudo-Voigt peak shape of the fit shape function [1,2], The quality of the fit, indicated by χ^2 values of 2.705 and 2.730 for XRD profiles at 25℃ and 250℃, respectively, demonstrates excellent agreement between the refined model and the experimental diffraction data.

Calculation of band gap

The band gap of the Cr-doped layered ceramic scaffold is calculated from UV-vis absorption based on Tauc's plots as following:

$$(\alpha hv)^{1/n} = B(hv - E_g)$$

Where, α is the absorption coefficient, h is the Planck constant, v is the light frequency, B is a constant, and E_g is the band gap. For direct band gap, n = 1/2, while n = 2 for indirect band gap. Therefore, the band gap can thus be obtained from the plots of $(\alpha hv)^{1/n}$ and photon energy hv.

Mechanical testing

Three-point bending strength, modulus and fracture toughness of nacre-mimetic composite and corresponding comparative materials were tested by Instron 5565A equipped with 500 N load cells. For bending test, the specimens were cut into ~2 mm in width, ~2 mm in depth and ~30 mm in length. The experimental loading rate was 1 μm/s with span of 12 mm. For single-edge notched bend (SENB), the specimens were

prepared into ~2 mm in width, ~4 mm in depth and ~30 mm in length with support span of ~16 mm, which complied ASTM E1820 measurement standard. In addition, the specimens of SENB were approximately 50% of its depth by using a 250 μm diamond saw and sharpened by razor blade. The loading rate also was 1 μm/s for the quasi-static mechanical test. The CE specimens were ~12.7 mm in width, ~2 mm in depth and span of ~32 mm, as referred to ASTM D790 for three-point bending tests. For in situ SENB specimens at 10 μm/s on optical microscope, the size was 2x2x15 mm. The strain distribution measured with DIC. This test was conducted in a mechanical testing machine (MTS Landmark 370.50) equipped with 20 KN load cell at rate of 1 μm/s and span of 64 mm. The SENB specimens were 8x16x100 mm. A speckle pattern was applied to the samples with a paint spray before testing. Using the high-speed camera (ARAMIS 3D & HHS) acquired images during the mechanical loading process. The analysis was performed using the digital image correlation software (GOM Correlation), carried out with a subset size and step size of 13 and 8, respectively. The environmental three-point bending strength was tested by a universal material testing machine (Shimadzu AGX-V) equipped with an environmental chamber (Shimadzu TCE-N350SD).

Mechanical calculation

The fracture toughness, K_{Ic}, of NACs, Al_2O_3 ceramics and CE was calculated used following equations [3].

$$K_{\mathrm{Ic}} = \frac{P_{\mathrm{Ic}}S}{BW^{\frac{3}{2}}} f(\frac{a}{W})$$

$$f\left(\frac{a}{W}\right)=\frac{3(\frac{a}{W})^{1/2}[1.99-\frac{a}{W}(1-\frac{a}{W})(2.15-\frac{3.93a}{W}+\left(\frac{a}{W}\right)^{2}]}{2(1+\frac{2a}{W})(1-\frac{a}{W})^{3/2}}$$

From the formula, P_{Ic} is the maximum load in the fracture toughness test of NACs, Al_2O_3 ceramics and CE, *S* is support span, *B* and *W* are respectively the thickness and width of the specimens, and *a* represents the notch depth.

Fracture toughness, K_{Jc}, is related to elastic and plastic contribution, which is connected to J-integral calculation.

$$J=J_{\mathrm{el}}+J_{\mathrm{pl}}$$

J_{el} is contributed by the elasticity which is account for linear elastic fracture mechanics.

$$J_{\mathrm{el}}=\frac{{K_{\mathrm{Ic}}}^{2}}{E'}$$

J_{pl} is contributed by the plasticity which is calculated with following equation. A_{pl} is the plastic area underneath the load-displacement curves in SENB tests.

$$J_{\mathrm{pl}}=\frac{2A_{\mathrm{pl}}}{B(W-a)}$$

K values can be transformed from *J* values by the following formula. Where, *E* is Young’s modulus, and *v* is the Poisson’s ratio.

$$K_{\mathrm{Jc}}=(JE')^{1/2}$$

$$E'=E/(1-v^{2})$$

Crack extension, Δa, was calculated according to previously reported equations as following.

$$a_n=a_{n-1}+\frac{W-a_{n-1}}{2}\frac{C_n-C_{n-1}}{C_n}$$

$$C_n=u_n/f_n$$

$$\Delta a = a_n - a$$

a_n and C_n are the crack length and complaisance respectively which are calculated at each point after the departure of creak. u_n and f_n are the displacement and force at each point after departure of the crack respectively. W is the width of the testing specimens.

Supplementary Figures

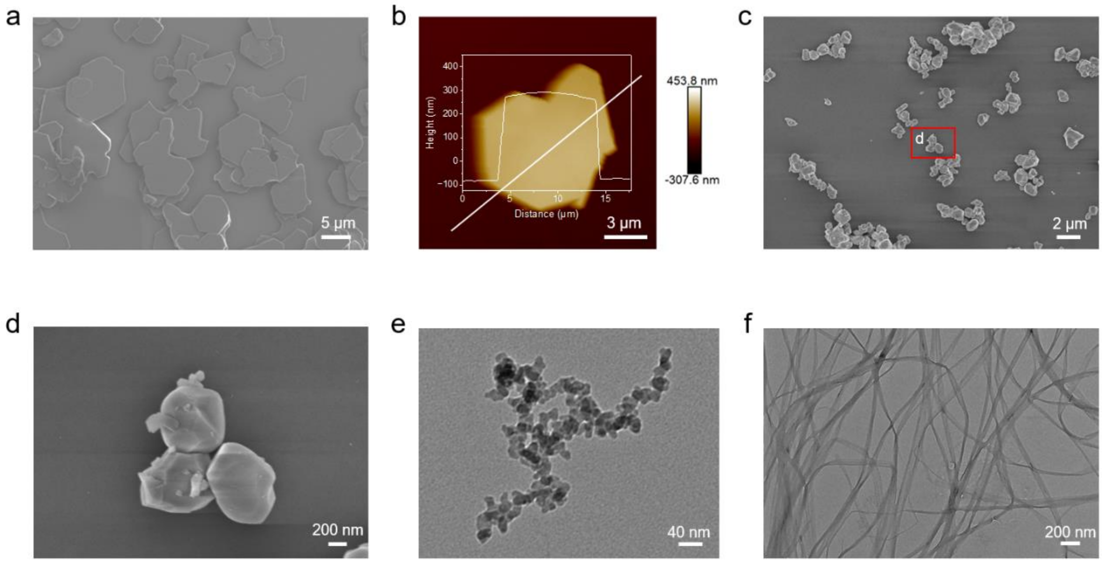

Figure S1. Characterization of assembly ingredient materials. (a) SEM image of Al_2O_3 MPs shows the micro-sized platelets. (b) AFM image of an Al_2O_3 MP shows the thickness (~400 nm). (c) SEM image of Cr_2O_3 NPs. (d) Magnified SEM image of (c). (e-f) TEM images of SiO_2 NPs (e) and BCNFs (f).

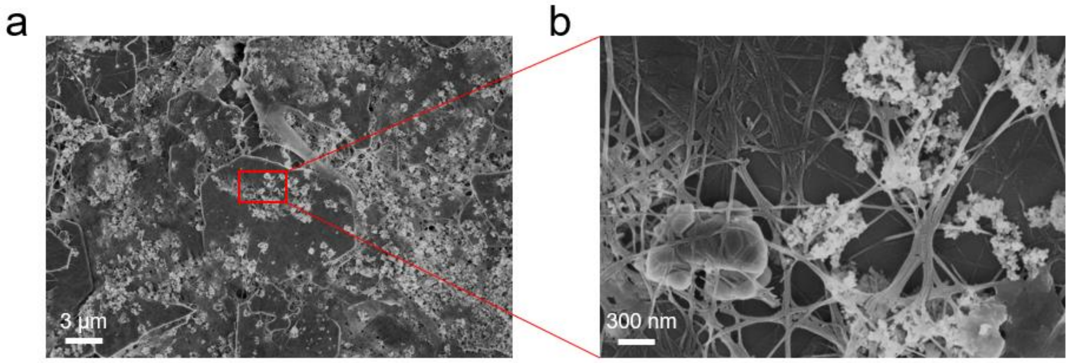

Figure S2. SEM images of layered nanocomposite films. (a) SEM image of the nanocomposite film surface showing Al_2O_3 MPs twined by BCNFs to form a layered structure. (b) Magnified SEM image of (a) shows that NPs are anchored to Al_2O_3 MPs by BCNFs.

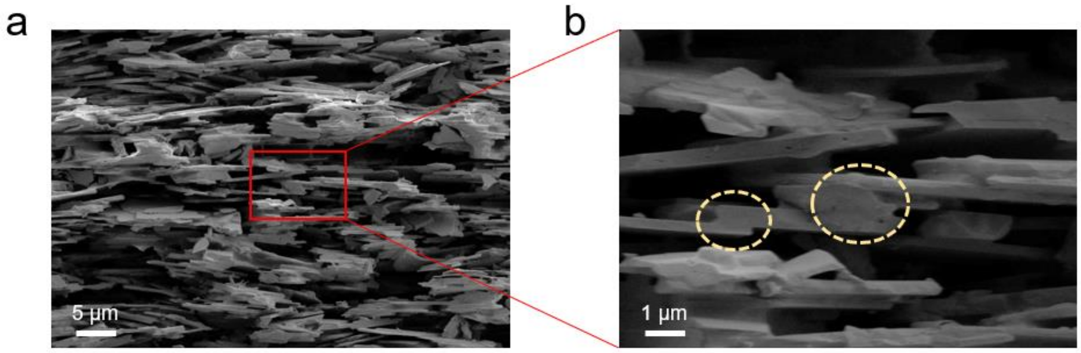

Figure S3. Microstructure of the layered ceramic scaffold. (a) SEM image shows that Al_2O_3 MPs are arranged in parallel after sintering. (b) Magnified SEM image of (a) shows the Al_2O_3 MPs are connected by mineral bridges (marked in yellow circles) formed from the NPs by sintering.

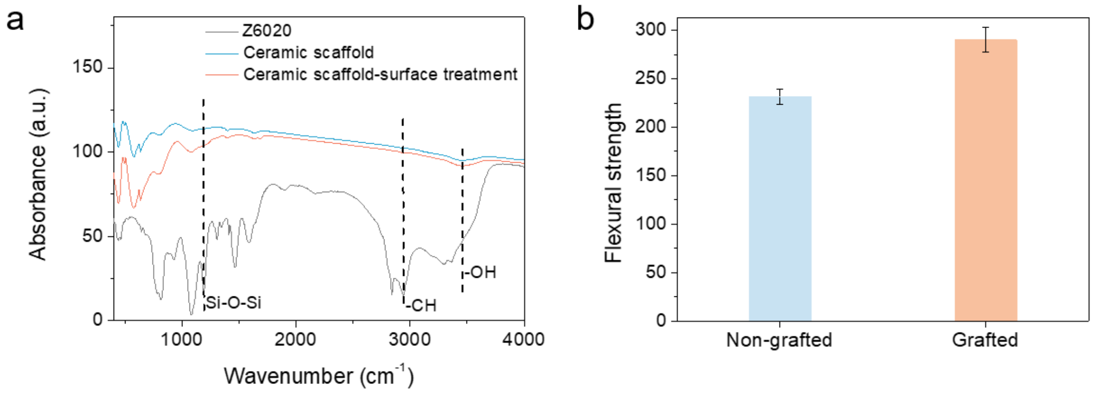

Figure S4. Layered ceramic scaffolds surface treatment. (a) FTIR spectra of Z6020 and the surface treatment ceramic scaffold utilizing Z6020. (b) Comparing flexural strength of NACs pre- and post- surface treatment. Chemical grafting increases the adhesion at the interface of organic and inorganic phase to enhance strength.

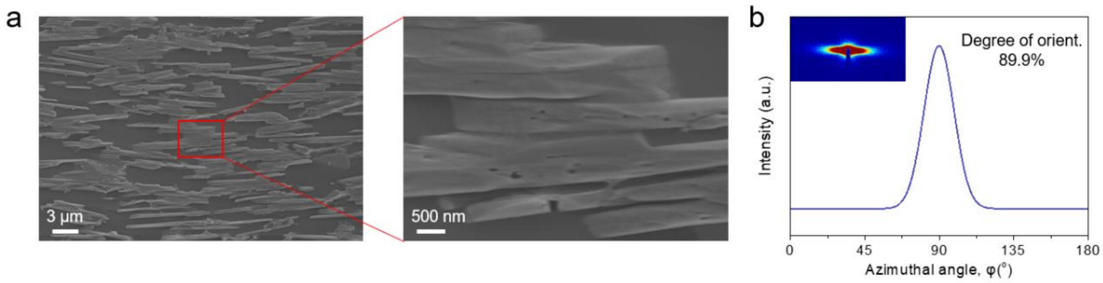

Figure S5. Microstructure observation of NACs. (a) SEM images of the NAC. (b) 2D SAXS image and azimuthal angle plot of the NAC.

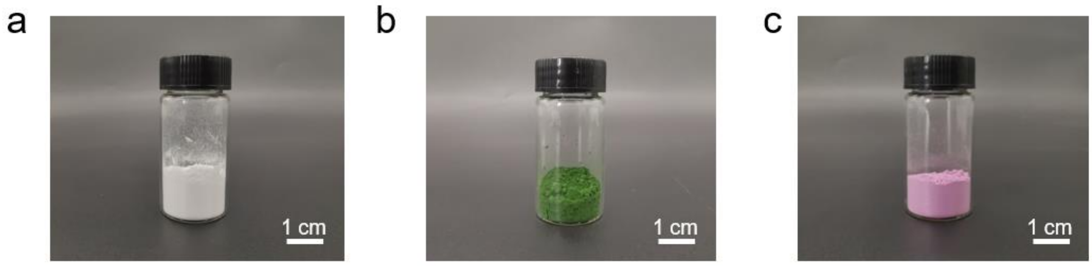

Figure S6. Color observation. Al_2O_3 MPs and Cr_2O_3 NPs (weight ratio 1:0.1) annealed at 1500℃. (a-c) Photographs of Al_2O_3 MPs (a), Cr_2O_3 NPs (b) and Cr-doped Al_2O_3 MPs prepared by 1500℃ annealing (c).

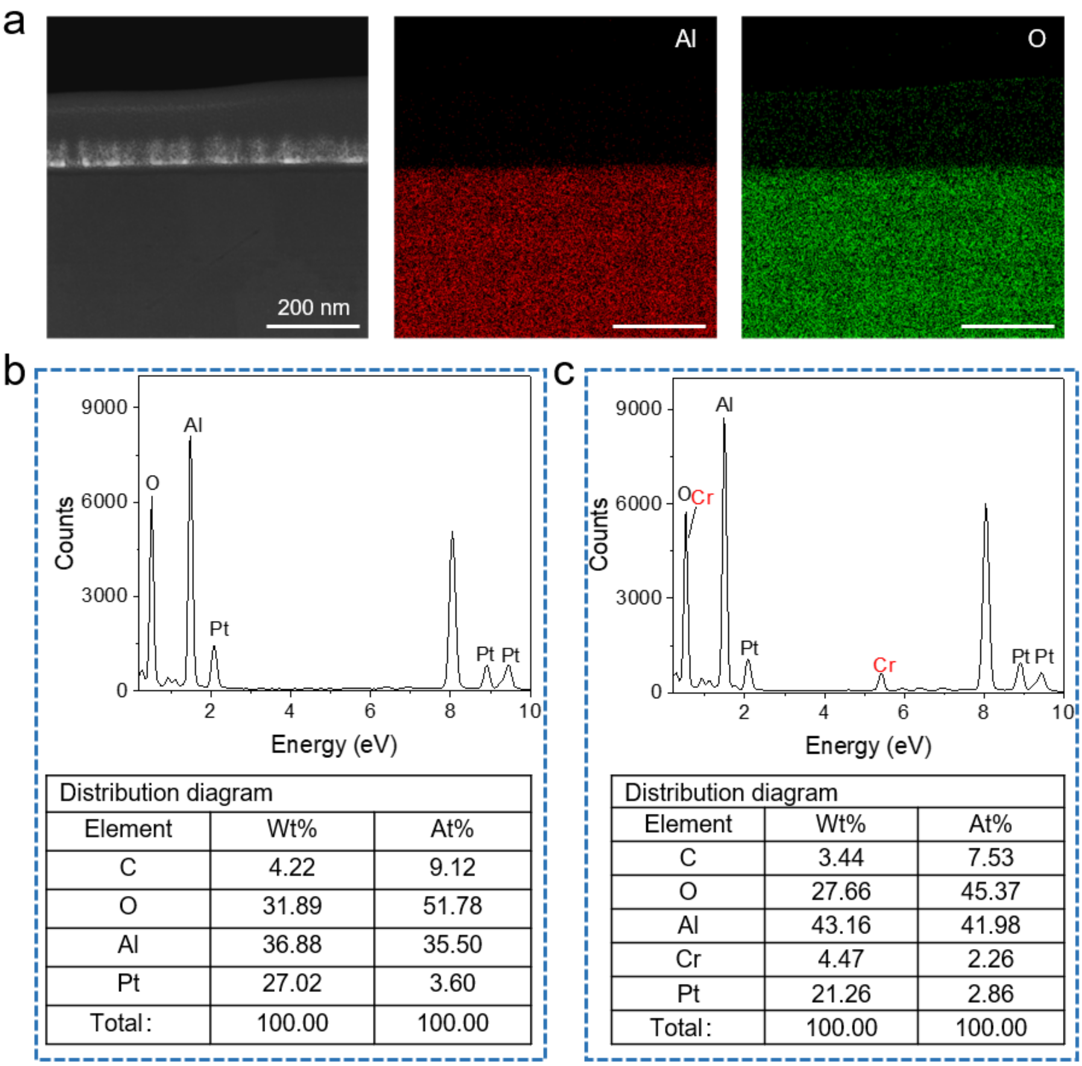

(b) Distribution diagram

Element	Wt%	At%
C	4.22	9.12
O	31.89	51.78
Al	36.88	35.50
Pt	27.02	3.60
Total:	100.00	100.00

(c) Distribution diagram

Element	Wt%	At%
C	3.44	7.53
O	27.66	45.37
Al	43.16	41.98
Cr	4.47	2.26
Pt	21.26	2.86
Total:	100.00	100.00

Figure S7. Elemental characterization. (a) TEM elemental maps of the Al_2O_3 MP before annealing. (b-c) Element distribution spectra of the Al_2O_3 MP before annealing (b) and the Cr-doped Al_2O_3 MP (c).

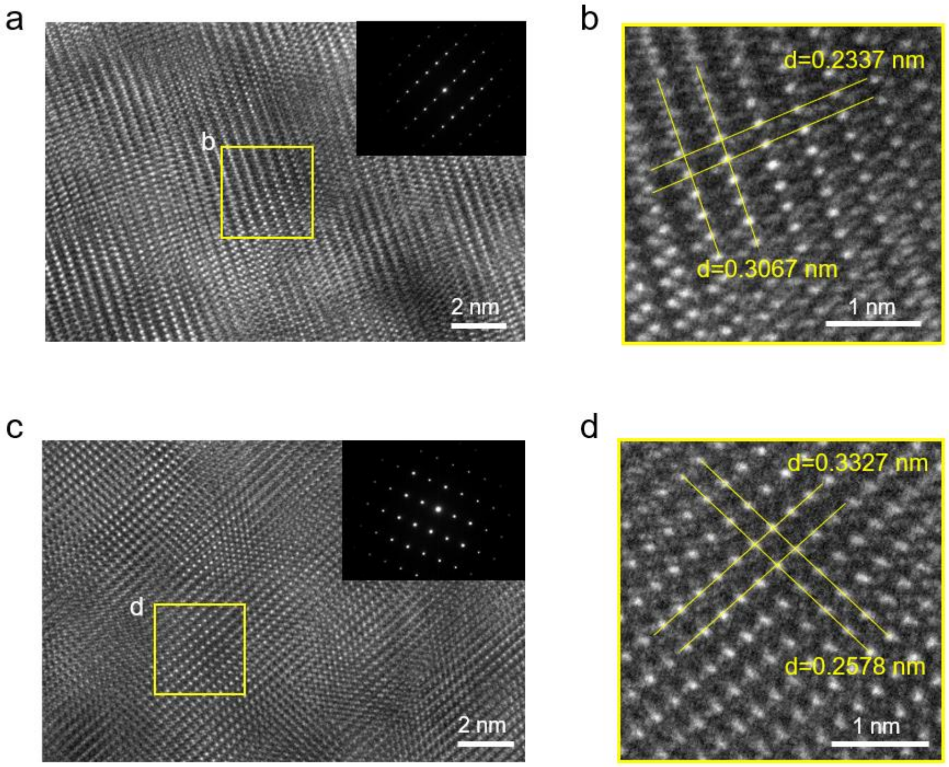

Figure S8. Crystal lattice observation. (a-b) HRTEM images of the Al_2O_3 MP before annealing. (c-d) HRTEM images of the Cr-doped Al_2O_3 MP. The illustrations in the upper right corner are the corresponding selected area electron diffraction (SAED) patterns. These results demonstrate the crystal lattice of Al_2O_3 becoming larger with Cr atoms doping.

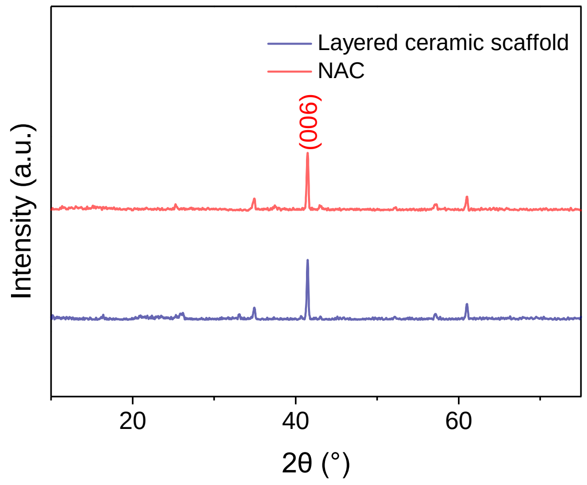

Figure S9. HRXRD patterns of the ceramic scaffold and NAC. Place the samples parallel to the ordered Al_2O_3 MPs in-plane direction on the test stage. The direction of Al_2O_3 MPs in-plane shows the intensity of (006) peak which demonstrates the crystal plane perpendicular to the optical axis of Al_2O_3 crystal.

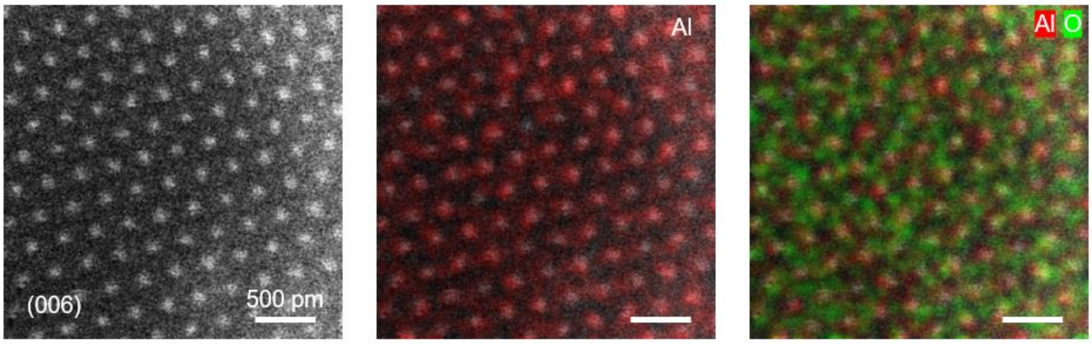

Figure S10. AC HAADF-STEM image and the corresponding EDS mapping of the Al_2O_3 MP without Cr-doped. HAADF-STEM image and atomic-resolution elemental maps of Al and O, viewed on the (006) crystal planes perpendicular to the optical axis of the Al_2O_3 single crystal.

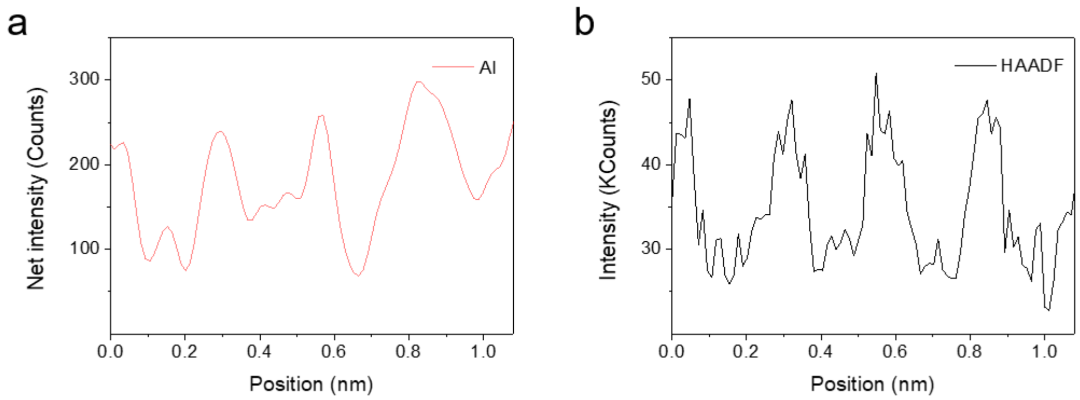

Figure S11. Intensity profiles of Fig. 2c along the white line. (a-b) Intensity profiles of elemental Al (a) and HAADF signal (b).

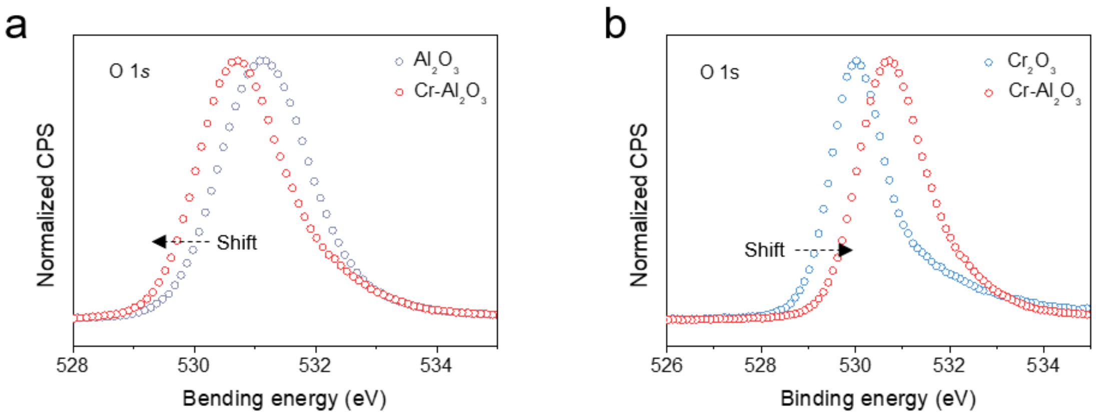

Figure S12. High-resolution XPS spectra of Cr-doped Al_2O_3 MPs. (a-b) Comparing O 1*s* of Cr-doped Al_2O_3 MPs with Al_2O_3 MPs (a) and Cr_2O_3 NPs (b), respectively.

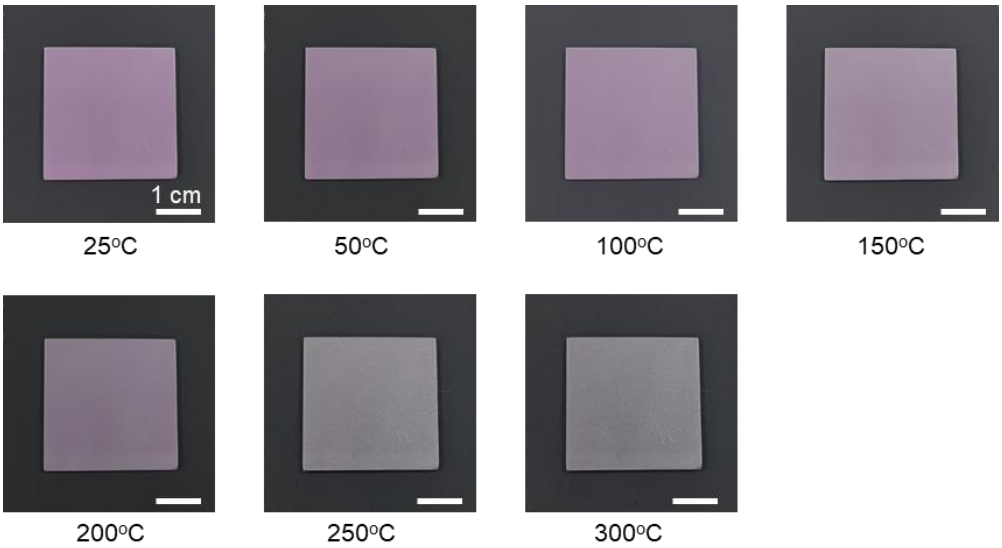

Figure S13. Photographs of the layered ceramic scaffold. These photos show the color change of the ceramic scaffold from 25℃ to 300℃.

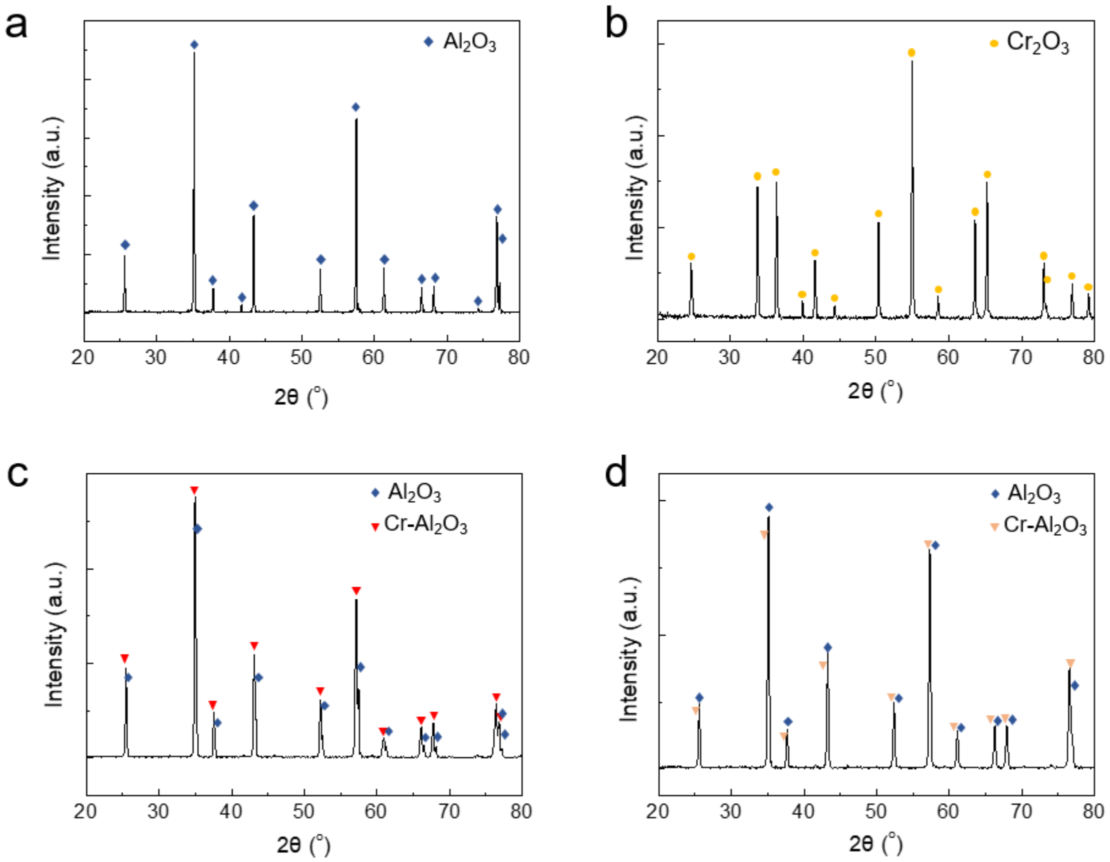

Figure S14. XRD spectra of inorganic minerals of ingredient materials and fabricated materials. (a-d) XRD spectra of Al_2O_3 MPs (a), Cr_2O_3 NPs (b), Cr-doped Al_2O_3 MPs (c) and ceramic scaffold powders (d).

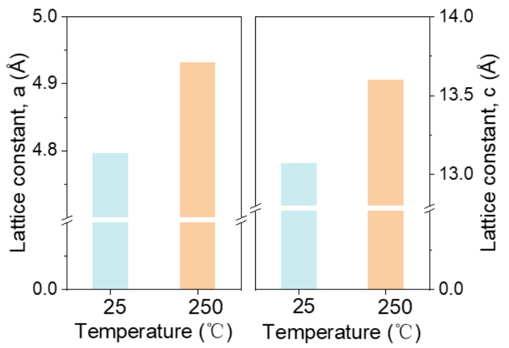

Figure S15. Crystal lattice parameters calculation. Al_2O_3 crystal in Cr-doped Al_2O_3 MPs expanded from 25℃ to 250 ℃.

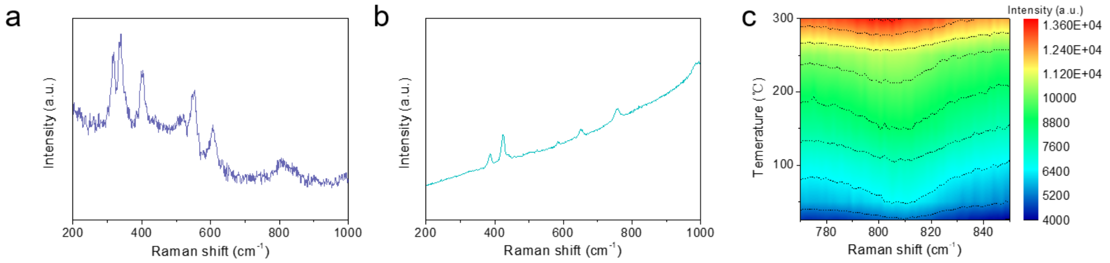

Figure S16. Raman spectra characterization. (a-b) Raman spectra of Cr_2O_3 NPs (a) and ceramic scaffolds without doped Cr_2O_3 NPs (b). (c) In situ heated Raman contour map of the Cr-doped ceramic scaffold.

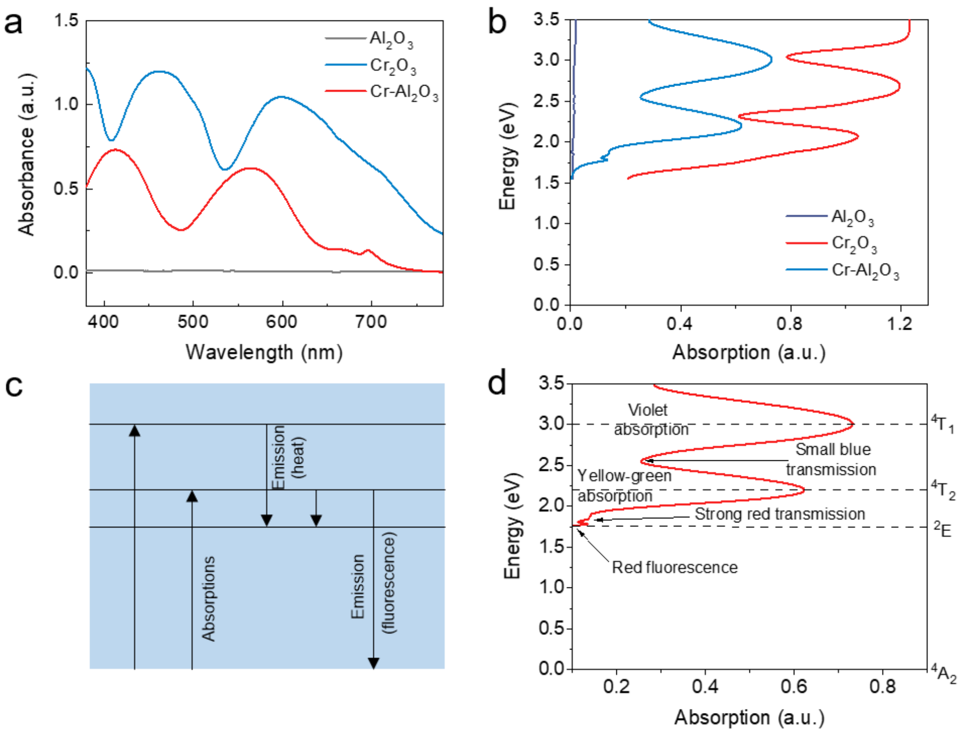

Figure S17. UV-Vis spectra characterization. (a) UV-Vis absorption spectra of Al_2O_3 MPs, Cr_2O_3 NPs and Cr-doped Al_2O_3 MPs. (b) Energy levels spectra calculated from (a) (E=1240/λ, E is energy, λ is wavelength). (c) Energy levels and transitions of Cr-doped Al_2O_3 MPs. (d) The resulting absorption spectra and fluorescence of Cr-doped Al_2O_3 MPs.

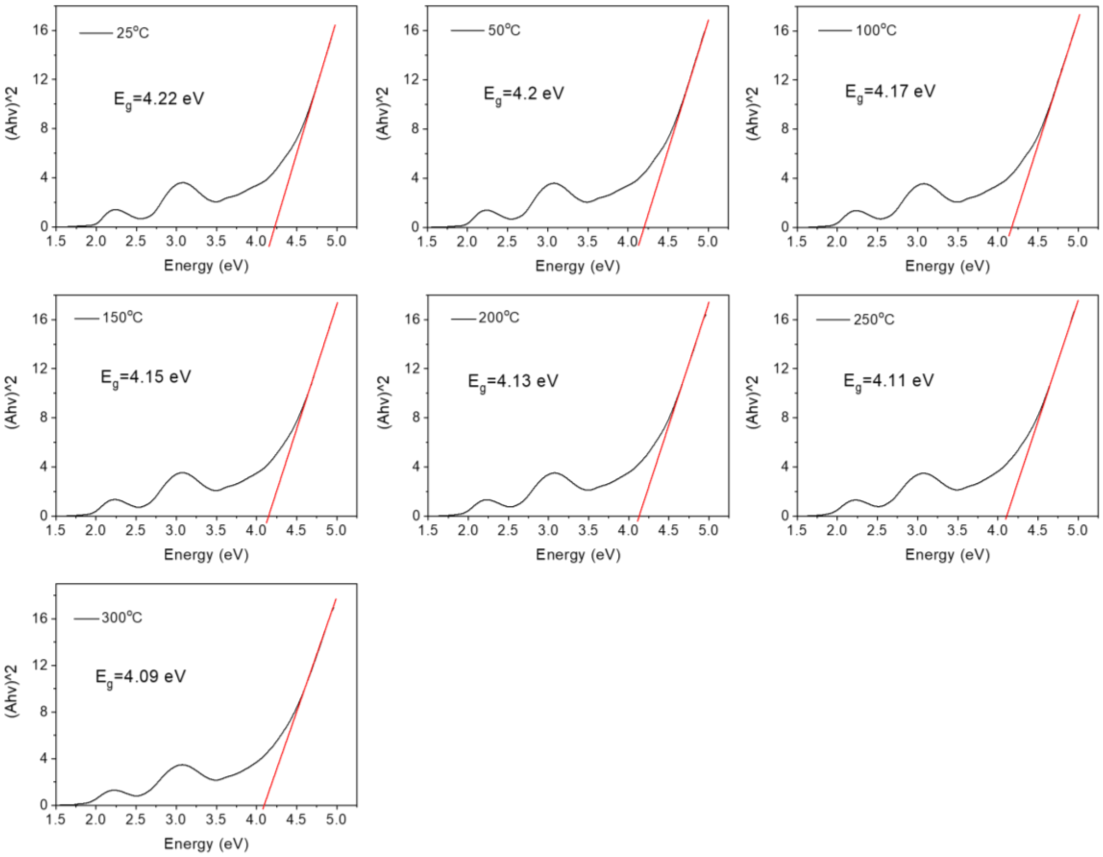

Figure S18. Absorption spectra band gap calculation. The corresponding Tauc's plots of the Cr-doped layered ceramic scaffold display the extrapolated optical band gaps for a heating process.

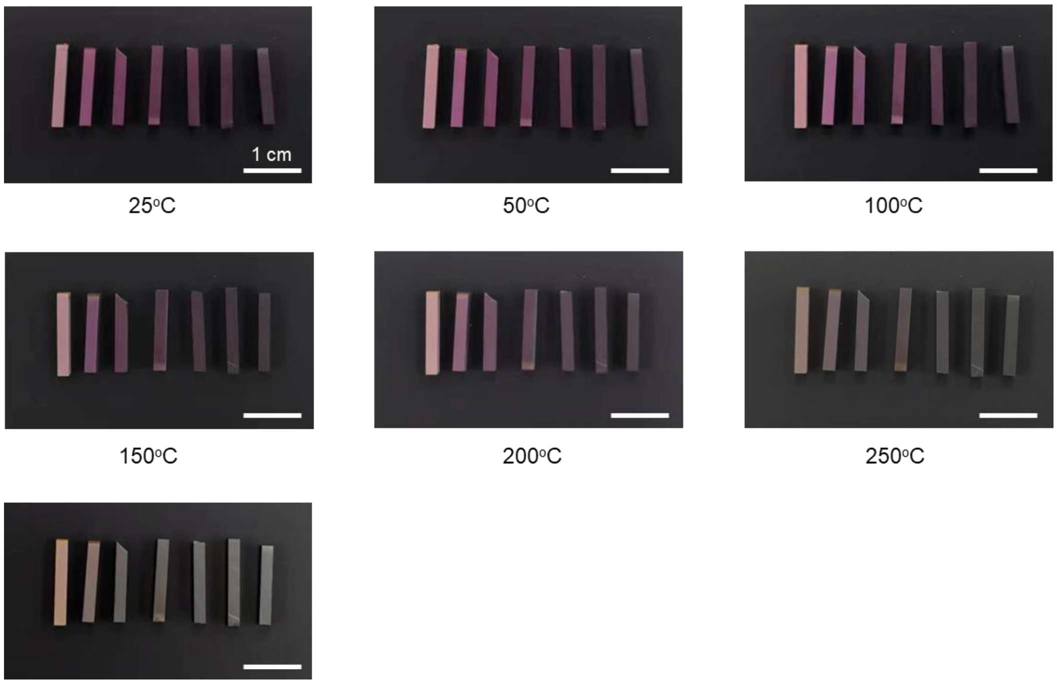

Figure S19. Photographs of biomimetic composites with different weight ratio of Cr_2O_3 NPs doping content during heating process. The weight ratio of SiO_2 NPs and Al_2O_3 MPs is kept unchanged at 0.1:1, and the weight ratio of Cr_2O_3 NPs to Al_2O_3 MPs from left to right is 0.05:1, 0.1:1, 0.15:1, 0.2:1, 0.25:1, 0.3:1, respectively.

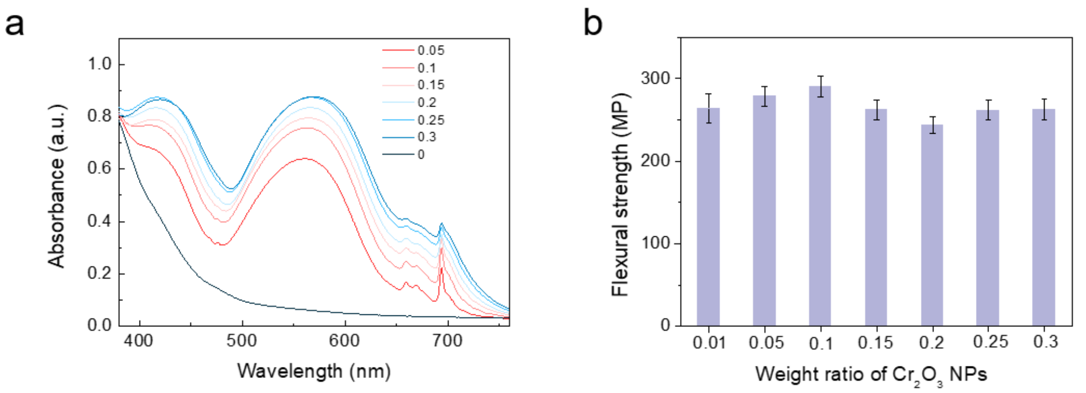

Figure S20. Optical and mechanical properties of biomimetic bulk composites with different weight ratio of Cr_2O_3 NPs. Keeping the weight ratio of SiO_2 NPs and Al_2O_3 MPs at 0.1:1 unchanging, increasing Cr_2O_3 NPs and Al_2O_3 MPs weight ratio. (a-b) UV-Vis spectra (a) and flexural strength (b) of biomimetic composites with different weight ratio of doped Cr_2O_3 NPs.

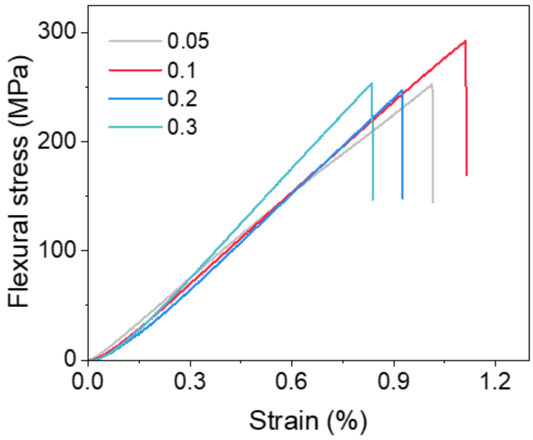

Figure S21. Flexural stress-train curves of biomimetic composites with different weight ratio SiO_2 NPs. Keeping the weight ratio of Cr_2O_3 NPs and Al_2O_3 MPs at 0.1:1, changing the weight ratio of SiO_2 NPs and Al_2O_3 MPs (0.05:1, 0.1:1, 0.2:1, 0.3:1).

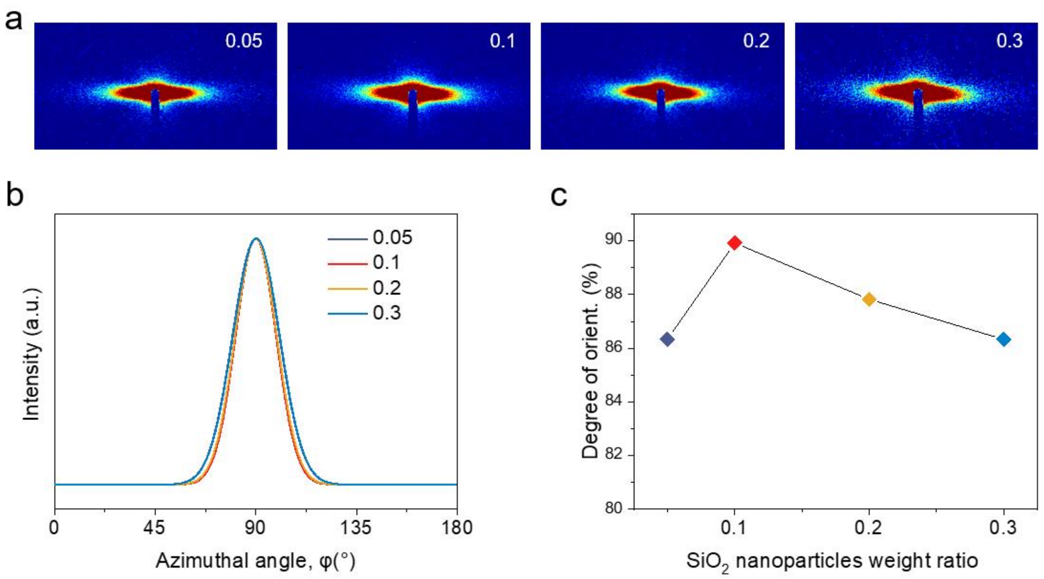

Figure S22. The ordering of Al_2O_3 MPs assembly in biomimetic composites. (a-b) 2D SAXS images (a) and azimuthal angle plots (b) of biomimetic composites with different weight ratio of SiO_2 NPs and Al_2O_3 MPs. (c) The degree of orientation of biomimetic composites with different SiO_2 contents.

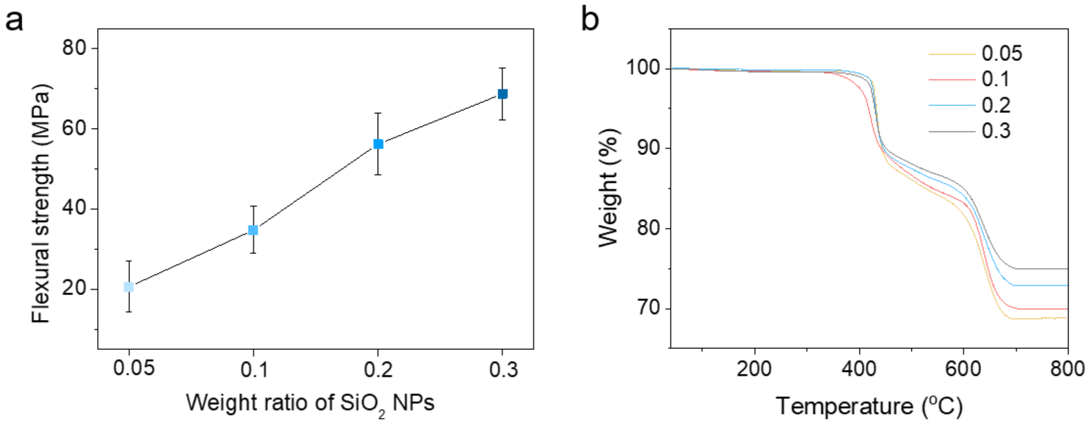

Figure S23. Inorganic component characterization of biomimetic composites. (a-b) Flexural strength of ceramic scaffolds (a) and TG curves of composites (b) with different weight ratio of SiO_2 NPs.

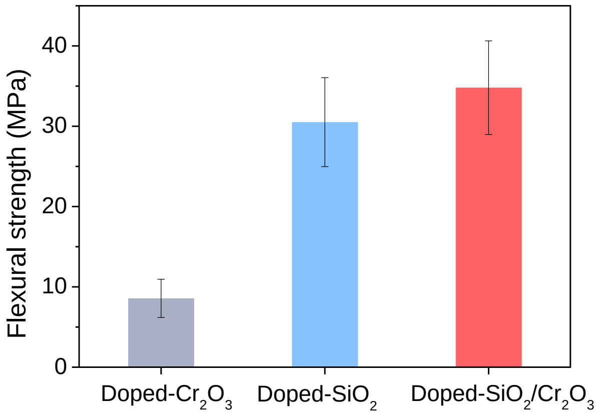

Figure S24. The mechanical effects of different NPs. Comparison of three-point bending strength of layered ceramic scaffolds with different kinds of NPs. The weight ratio as following, Al_2O_3 MPs: Cr_2O_3 NPs 1:0.1, Al_2O_3 MPs: SiO_2 NPs 1:0.1, Al_2O_3 MPs: SiO_2 NPs: Cr_2O_3 NPs 1:0.1:0.1, respectively.

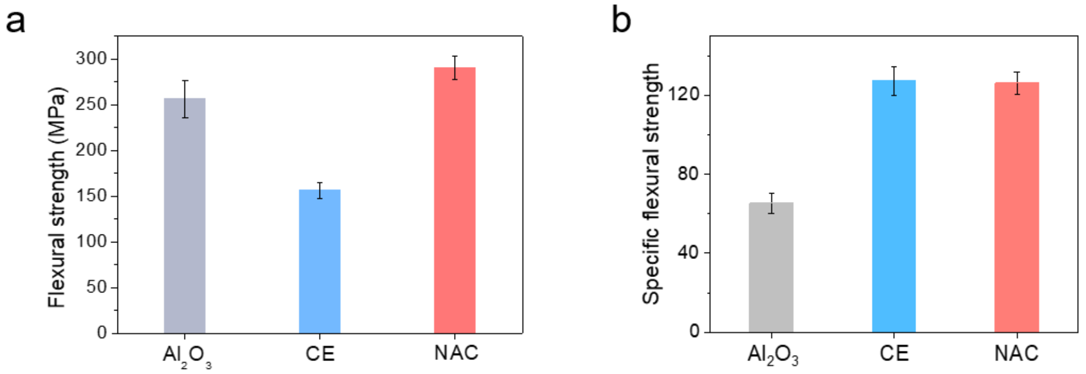

Figure S25. Comparison of mechanical strength of NACs with their component materials. (a-b) Comparing flexural strength (a) and Specific flexural strength (b) of the optimal NACs with Al_2O_3 ceramics and CE.

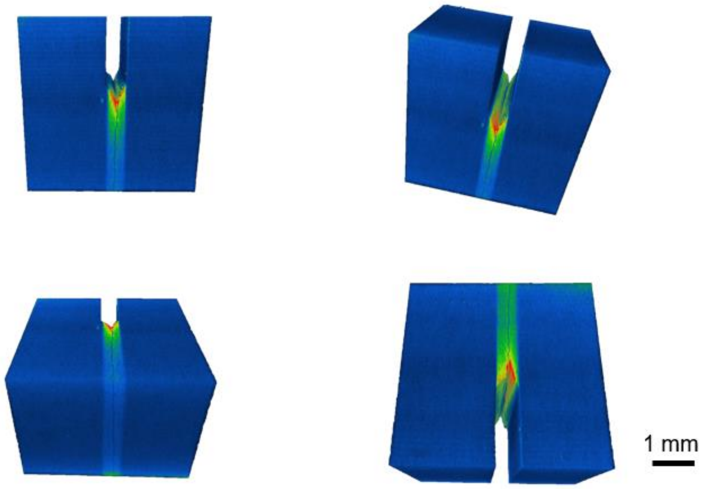

Figure S26. X-ray tomography images of the NAC. 3D reconstruction of the NAC following SENB test by micro-computed tomography technique.

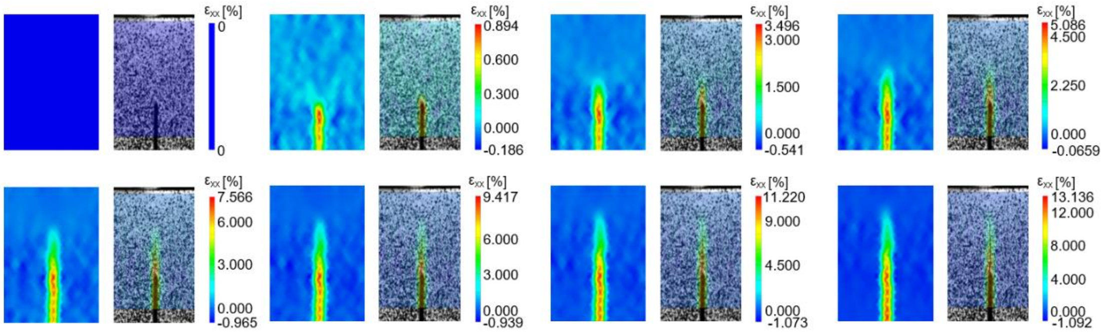

Figure S27. DIC maps of the CE during the crack propagation. There is large deformation in crack propagation but no crack deflection, which is attributed to its isotropic structure without preventing crack growth.

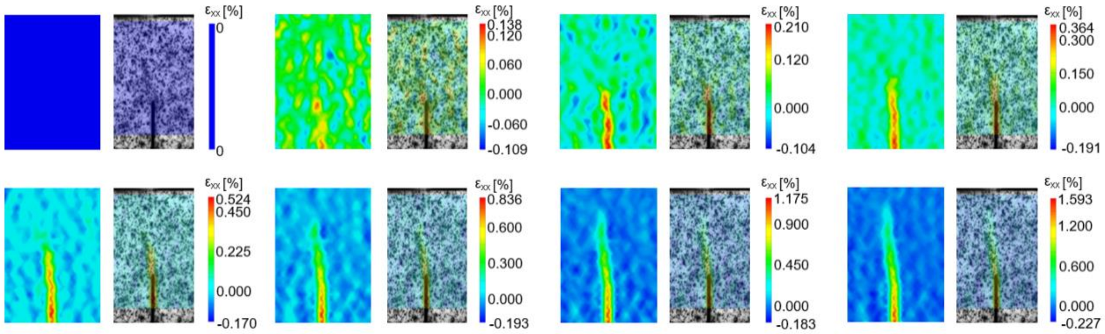

Figure S28. DIC maps of the Al_2O_3 ceramic during the crack propagation. There is minimal deformation and no crack deflection in the process of crack propagation, indicating its brittleness.

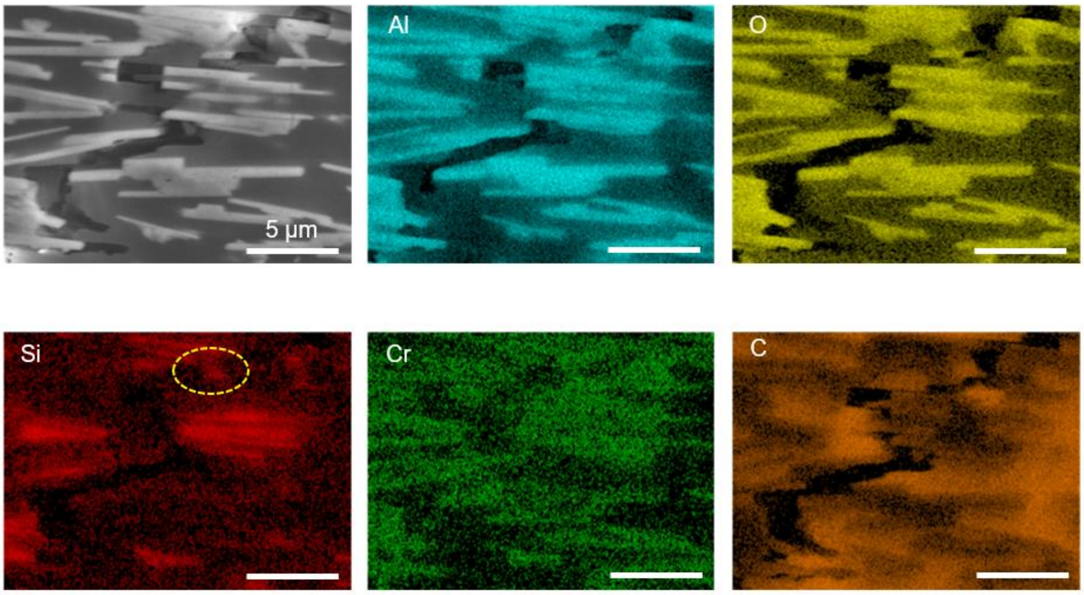

Figure S29. Elemental characterization of the bioinspired NAC crack. Crack propagation behaviors observed from EDS elemental maps mainly occurs in organic phase, but it can also be seen in the fracture of mineral bridges.

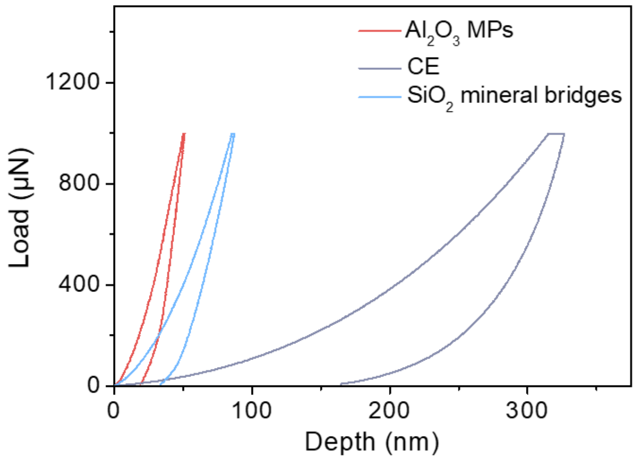

Figure S30. Load-depth curves of the NAC. The Load-depth curves of three main microstructures (Al_2O_3 MPs, polymer CE and inorganic mineral bridges SiO_2) were measured by nanoindentation test.

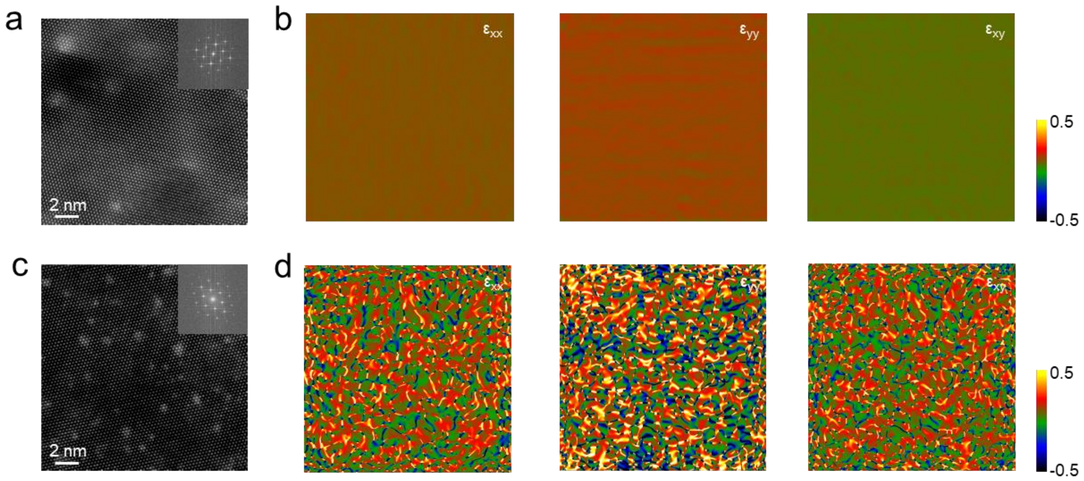

Figure S31. Determination of strain distribution by geometric phase analysis (GPA). (a-b) Atomic-scale HAADF-STEM image of Al_2O_3 crystal (a) and corresponding ε_{xx}, ε_{yy} and ε_{xy} strain maps (b) obtained by GPA. (c-d) Atomic-scale HAADF-STEM image of Cr-doped Al_2O_3 crystal (c) and corresponding ε_{xx}, ε_{yy} and ε_{xy} strain maps (d). The insets are the corresponding fast Fourier transform images.

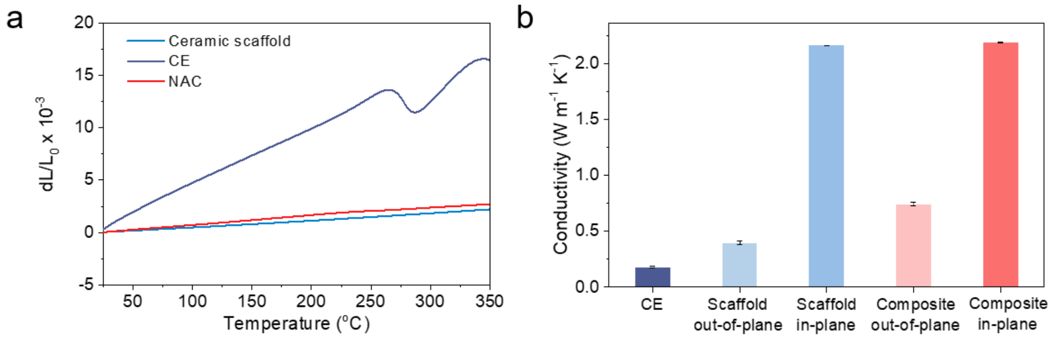

Figure S32. Comparison of thermal properties. (a) Thermal expansion of the NAC, CE and the layered ceramic scaffold. (b) Thermal conductivity of CE, out-of-plane and in-plane of ceramic scaffolds and NACs.

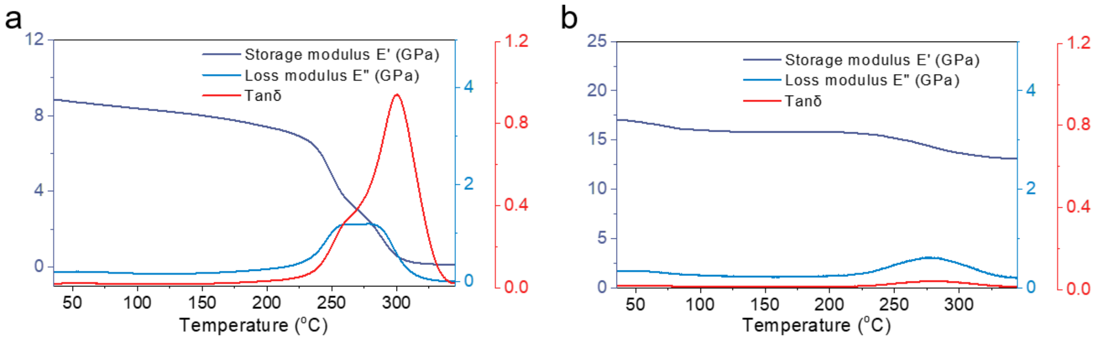

Figure S33. DMA characterization. (a-b) DMA of CE (a) and the NAC (b) with the temperature increasing.

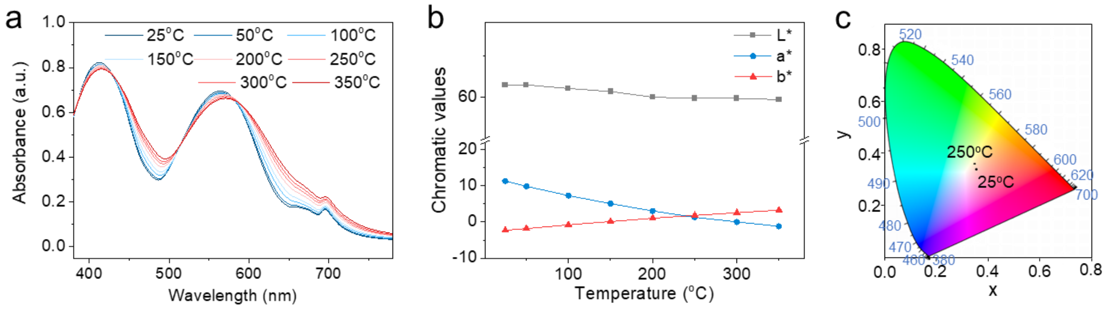

Figure S34. The color change of Cr-doped Al_2O_3 MPs. (a) UV-Vis absorption spectra of Cr-doped Al_2O_3 MPs with increasing temperature. (b) Chromatic values of Cr-doped Al_2O_3 MPs. (c) CIE chromaticity coordinates of Cr-doped Al_2O_3 MPs at 25℃ and 250℃ in the 1931 color space chromaticity diagram.

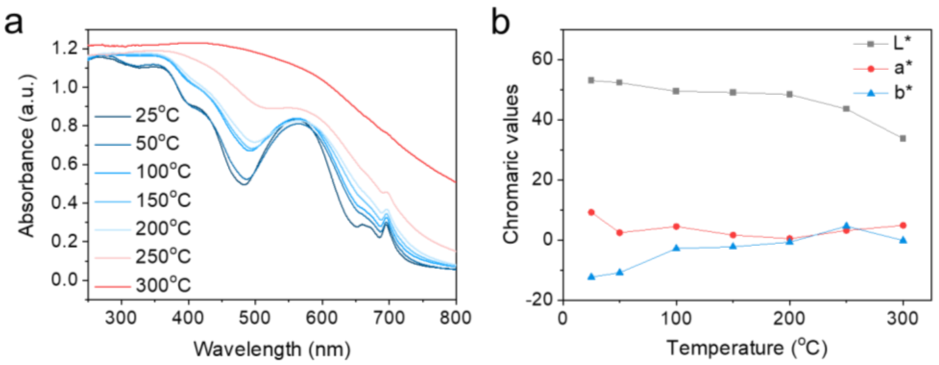

Figure S35. The color change of the NAC as temperature increasing. (a) UV-Vis absorption spectra of the NAC. (b) Chromatic values of the NAC.

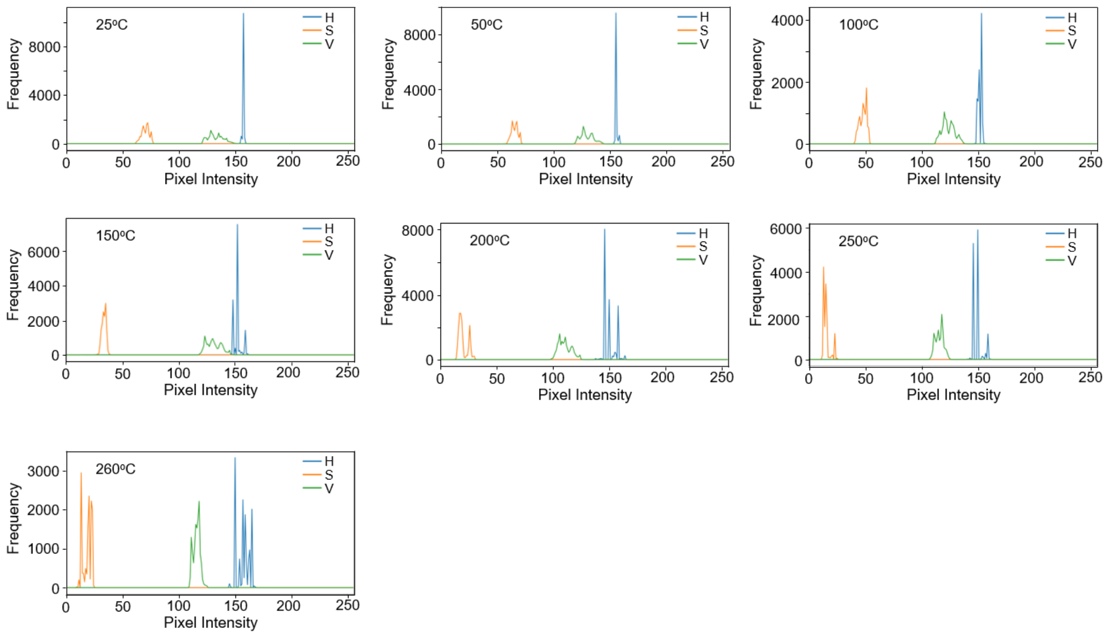

Figure S36. HSV features of the NAC under different temperatures. The alteration of color involves primarily changes in the S values and H values.

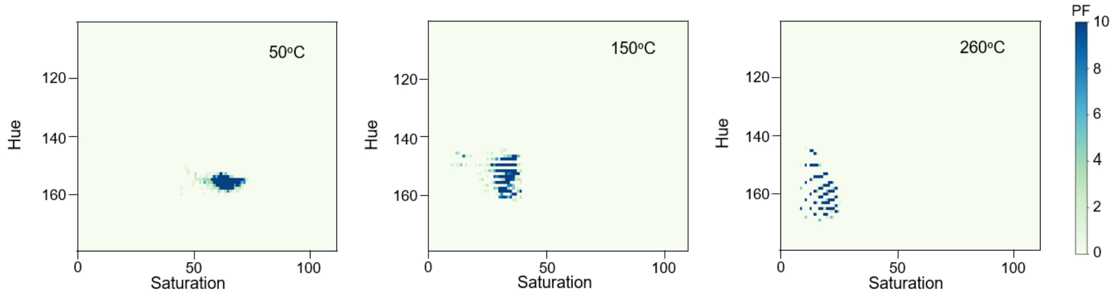

Figure S37. H-S 2D color histograms. The 2D histograms show the values of H-S at 50℃, 150℃and 260℃.

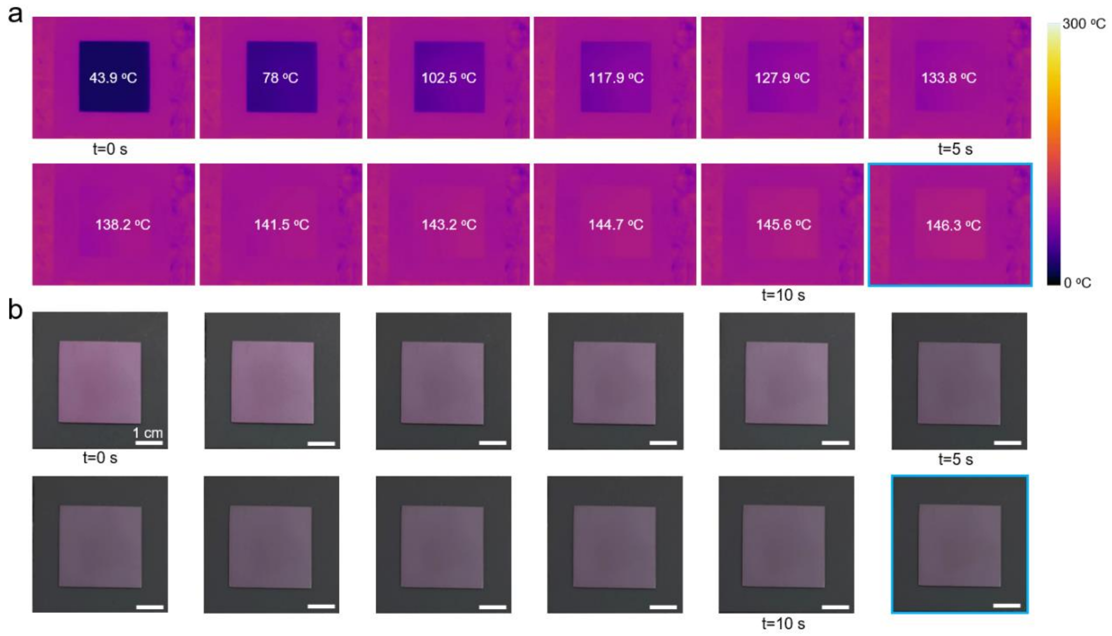

Figure S38. Temperature and color change on a 150℃ heating table of the NAC. (a) Infrared images at different heating times for verifying the accuracy of image recognition. (b) Photographs of the NAC at different heating times. The time corresponding to the image circled by the blue border represents the response time obtained through the trained image recognition program.

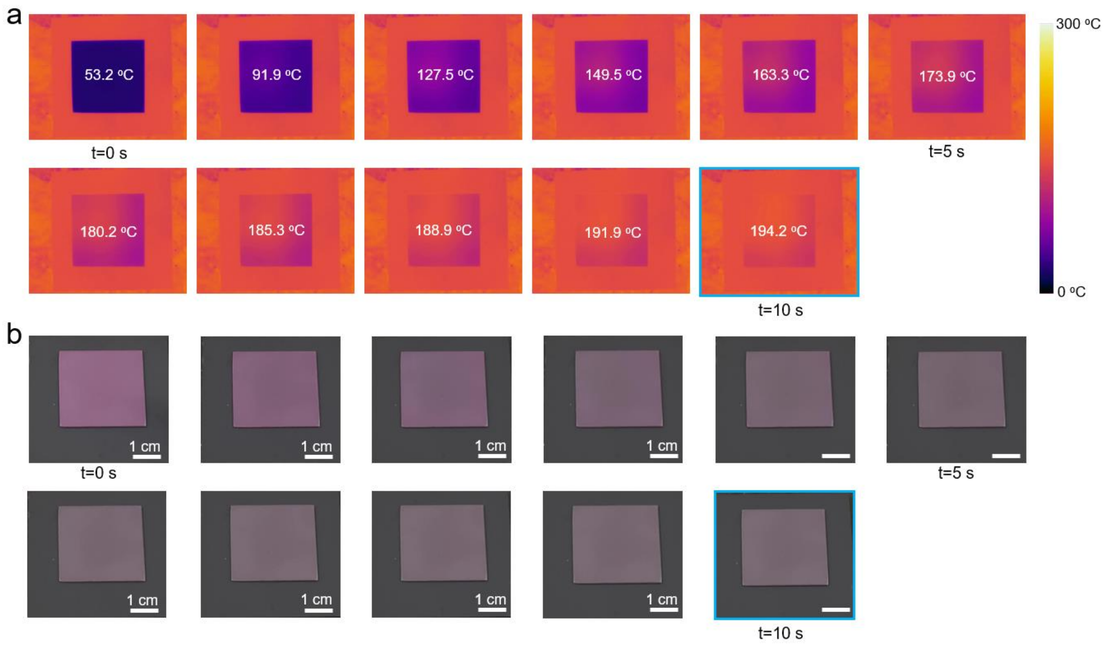

Figure S39. Temperature and color change on a 200℃ heating table of the NAC. (a) Infrared images at different heating times for verifying the accuracy of image recognition. (b) Photographs of the NAC at different heating times. The time corresponding to the image circled by the blue border represents the response time obtained through the trained image recognition program.

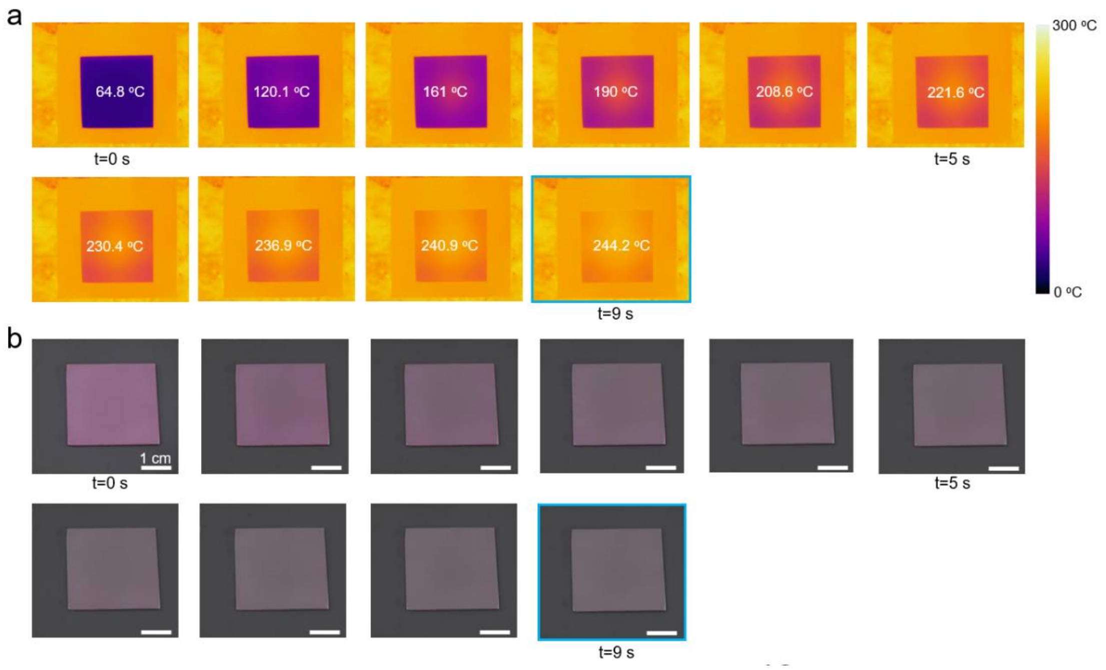

Figure S40. Temperature and color change on a 250℃ heating table of the NAC. (a) Infrared images at different heating times for verifying the accuracy of image recognition. (b) Photographs of the NAC at different heating times. The time corresponding to the image circled by the blue border represents the response time obtained through the trained image recognition program.

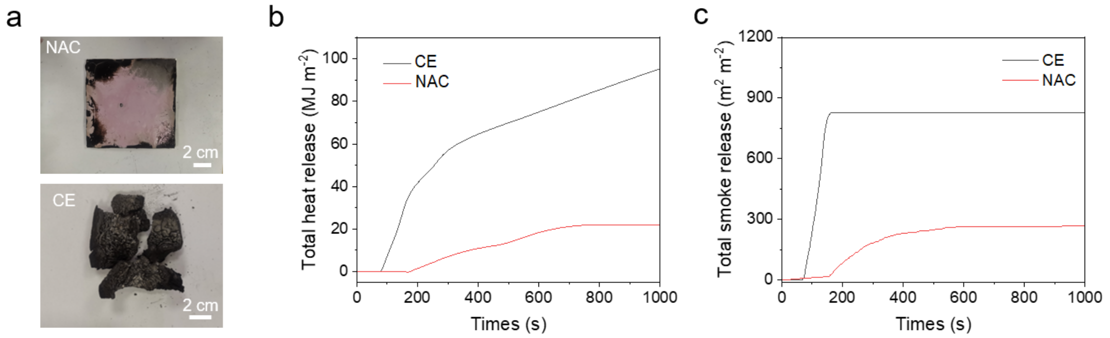

Figure S41. Cone calorimetry testing of NACs and CE. (a) Photographs the NAC and CE following Cone calorimetry testing. (b-c) Total heat release curves (b) and Total smoke release curves (c) of the NAC and CE.

Supplementary table

Table S1 Comparison of mechanical properties of NACs with a series of engineering materials.

Materials	Density (g cm^{-3})	Flexural strength (MPa)	Fracture toughness K_{Jc} (MPa m$^{1/2}$)	Specific strength (MPa/(Mg m^{-3}))	Specific toughness (MPa m$^{1/2}$/(Mg m^{-3}))	Reference
Cu-alloys	8.5	221 (s.d.=136)	56.53 (s.d.=29.3)	26 (s.d.=16)	6.55 (s.d.=3.45)	(Bouville et al., 2014)
Al-alloys	2.8	212.8 (s.d.=151)	30.25 (s.d.=11.5)	76 (s.d.=54)	10.9 (s.d.=4.1)	(Bouville et al., 2014)
Fe-alloys	-	-	-	39.5 (s.d.=20.5)	2.15 (s.d.=0.95)	(Bouville et al., 2014)
Concrete	2.4	12	0.8 (s.d.=0.6)	5 (s.d.=0)	0.33 (s.d.=0.25)	(Gao et al., 2017)
Silica glass	2.6	117 (s.d.=60)	0.6 (s.d.=0.1)	45 (s.d.=23.1)	0.23 (s.d.=0.04)	(Gao et al., 2017)
SiC	3.2	338 (s.d.=5)	3.5 (s.d.=1.0)	105.6 (s.d.=1.56)	1.09 (s.d.=0.31)	(Bouville et al., 2014)
Alumina	3.96	315 (s.d.=15)	3.9 (s.d.=0.6)	124 (s.d.=41)	1.3 (s.d.=0.24)	(Bouville et al., 2014)
ZrO_2	-	-	-	93.4 (s.d.=8.49)	1.02 (s.d.=0.51)	(Bouville et al., 2014)
This work	2.3	290.1 (s.d.=12.8)	11.1 (s.d.=0.5)	126.24 (s.d.=5.6)	4.818 (s.d.=0.2)	-

Movie S1. In-situ loading crack propagation of the Al_2O_3 ceramic.

Movie S2. In-situ loading crack propagation of the CE.

Movie S3. In-situ loading crack propagation of the NAC.

Movie S4. The NAC for fire warning.

References

1. Toby B. EXPGUI, a graphical user interface for GSAS. *J Appl Crystallogr* 2001; **34**: 210-3.

2. Finger LW, Cox DE, Jephcoat AP. A correction for powder diffraction peak asymmetry due to axial divergence. *J Appl Crystallogr* 1994; **27**: 892-900.

3. Gao H-L, Chen S-M, Mao L-B *et al.* Mass production of bulk artificial nacre with excellent mechanical properties. *Nat Commun* 2017; **8**: 287.
